# Investigation of *Fructus sophorae extract*’s therapeutic mechanism in atrophic vaginitis based on network pharmacology and experimental validation

**DOI:** 10.3389/fphar.2025.1571976

**Published:** 2025-05-08

**Authors:** Jiarui Zheng, Weiming Wang, Wenting Song, Yehao Zhang, Mingjiang Yao, Guangrui Wang, Zishan Huang, Feng Li, Junguo Ren, Li Lin, Xiaodi Fan, Jianxun Liu

**Affiliations:** ^1^ Institute of Basic Medical Sciences of Xiyuan Hospital, China Academy of Chinese Medical Sciences, Beijing Key Laboratory of Pharmacology of Traditional Chinese Medicine, Beijing, China; ^2^ Institute of Chinese Medicine, Heilongjiang Academy of Traditional Chinese Medicine, Harbin, China

**Keywords:** *Fructus sophorae extract*, atrophic vaginitis, network pharmacology, molecular docking, experimental verification

## Abstract

**Objective:**

This study systematically elucidates the therapeutic mechanism of *Fructus sophorae* extract (FSE) against atrophic vaginitis (AV) by integrating network pharmacology with *in vitro* experimental validation.

**Methods:**

Potential drug targets of FS and AV-related disease targets were systematically retrieved from TCMSP, SWISS Target Prediction, GeneCards, and DisGeNET databases. The putative therapeutic targets of FS against AV were identified through target overlap analysis between drug and disease targets. Functional enrichment analyses of GO terms, KEGG pathways, and disease associations were performed using DAVID database, with results visualized by Cytoscape software. Molecular docking validation and binding affinity visualization between FS components and target proteins were carried out using PubChem database and PyMOL software. The AV animal model was established by bilateral ovariectomy (OVX). To validate FS’s effects on target protein expression, immunohistochemical staining and Western blot analyses were performed.

**Results:**

Through target intersection analysis between 137 drug targets and 1,777 disease targets, a total of 100 potential therapeutic targets were identified for FS in AV treatment. Subsequent core gene screening revealed key targets, namely, EGFR, AKT1, ESR1, and TNF. GO and KEGG enrichment analyses demonstrated significantly enriched pathways, with the PI3K/AKT signaling pathway showing particular relevance. Molecular docking analysis revealed strong binding affinity between FS components and the functional domains of EGFR, AKT1, and ESR1. An OVX-induced rat AV model was successfully established, with pathological and molecular validation achieved via immunohistochemistry and Western blot analyses. FS treatment significantly normalized the dysregulated expression levels of p-PI3K/PI3K, p-AKT/AKT, ERα, EGF, and EGFR.

**Conclusion:**

FS demonstrates multi-target regulatory capacity, specifically modulating p-PI3K/PI3K, p-AKT/AKT, ERα, EGF, and EGFR signaling pathways, which substantiates its potential as a promising therapeutic agent for AV. These findings provide mechanistic insights into FS’s therapeutic targets against AV, establishing a theoretical foundation for its translational application in AV therapy.

## 1 Introduction

Atrophic vaginitis (AV), also referred to as Senile Vaginitis (SV), represents a prevalent condition that significantly affects women, especially postmenopausal individuals and cancer survivors with ovarian dysfunction. AV exhibits high prevalence rates, with epidemiological studies suggesting that >50% of postmenopausal women manifest symptoms characteristic of this condition (Benini et al., 2022; Chua et al., 2017). The characteristic clinical manifestations comprise vaginal dryness, mucosal irritation, pruritus, dysuria, and dyspareunia (Mac Bride et al., 2010; Cox et al., 2023). This rising prevalence reflects demographic aging and increasing iatrogenic ovarian failure cases, particularly following chemotherapy or bilateral oophorectomy. Such interventions can precipitate premature AV onset, thereby extending its clinical burden to premenopausal women otherwise unaffected by menopausal changes (Juhász et al., 2021). Current therapeutic strategies encompass estrogen replacement therapy, vaginal *lactobacillus* supplementation, topical metronidazole, vaginal pH normalization procedures, and traditional Chinese medicine formulations (e.g., Baofukang suppositories) (Hainer and Gibson, 2011). However, these interventions primarily provide symptomatic relief rather than disease-modifying effects specific to AV pathogenesis. Clinical underreporting remains prevalent due to patients’ concerns regarding estrogen therapy safety, often leading to delayed treatment and disease progression (Ulhe et al., 2024). Therefore, developing pathogenesis-targeted therapies constitutes a critical unmet need in AV management.

Although estrogen replacement therapy has shown therapeutic efficacy for AV, its clinical application is limited by adverse effects (Lester et al., 2015). Therefore, phytochemicals have gained attention as potential alternatives (Mazalzadeh et al., 2020). These plant-derived compounds demonstrate favorable safety profiles and reduced toxicity compared to synthetic drugs. Moreover, they exhibit multi-target modulatory properties that may attenuate disease progression (Patti et al., 2018). Thus, select phytochemicals represent promising candidates for AV therapy. AV. *Fructus sophorae* (FS) contains a complex phytochemical profile, featuring bioactive compounds including genistein and rutin. These components demonstrate estrogen-like activity, rendering them particularly relevant for managing postmenopausal osteoporosis resulting from estrogen deficiency (Abdallah et al., 2014). Accumulating evidence indicates that FS flavonoids not only display estrogenic effects (Kim, 2016),but also exert anti-inflammatory (Jiang et al., 2018), anti-osteoporotic (Chakuleska et al., 2022), and anti-neoplastic activities (dos Santos et al., 2023). Sophoroside (Soph), the predominant isoflavone glycoside in FS, mediates many of its pharmacological effects. This compound demonstrates multi-pharmacological properties encompassing anti-fertility, anti-allergic, anti-inflammatory, anti-hypertrophic, and lipid-modulating activities (Liu et al., 2022).

The rapid advancement of bioinformatics has established network pharmacology as a powerful approach that leverages large-scale databases to systematically elucidate pharmacological mechanisms at molecular and pathway levels (Guo et al., 2019). This methodology is particularly suited for investigating multi-target agents (Lee et al., 2019), as it integrates multidisciplinary approaches such as systems biology, polypharmacology, computational bioinformatics, and molecular dynamics simulations. Numerous studies have successfully applied network pharmacology to decipher drug-disease interactions across various pathological conditions. Moreover, this strategy represents a transformative paradigm for accelerating drug discovery processes (Wu et al., 2022). However, no comprehensive network pharmacology studies have been conducted to investigate FS in the context of AV to date. This study aimed to integrate network pharmacology with molecular docking to identify potential therapeutic targets, characterize biological functions, and delineate signaling pathways associated with FS’s therapeutic effects against AV. To experimentally validate the *in silico* predictions, we assessed FSE’s effects on target protein expression using an OVX-induced AV rat model. These findings provide novel mechanistic insights and preclinical evidence supporting FS’s potential as a therapeutic agent for AV.

## 2 Materials and methods

### 2.1 Material

#### 2.1.1 Test drug

*Fructus sophorae* extract (FSE), containing >92% sophoraside, was prepared as 0.2 g/grain vaginal suppositories for rat administration (batch number: 20230601), provided by the Heilongjiang Academy of Traditional Chinese Medicine. Following established protocols (Sun et al., 2019; Xu and Wang, 2022), 20 g of Fructus sophorae powder was subjected to ethanol reflux extraction (80% v/v, 8:1 solvent-to-material ratio, 40 min) to yield a sophoraside-rich ethanolic extract. The crude extract was then purified by macroporous resin chromatography to obtain FSE with 92% sophoraside content. For suppository preparation, PEG-6000 and glycerol were added as excipients and hot-melted (70°C) to fabricate 0.2 g/grain suppositories containing FSE at graded concentrations. Additionally, Promestriene cream (batch number: 230302) was obtained from Jiangxi Decheng Pharmaceutical Co., Ltd.

#### 2.1.2 Animals

Eighty-five female Sprague-Dawley (SD) rats (8-week-old, 200–220 g) were procured from Huafukang Biotechnology Co., Ltd. The animal production certificate number is SCXK (Jing) 2019-0008. Environmental parameters were controlled at 23°C ± 1°C, 55% ± 10% relative humidity, with all animals maintained under 12:12 h light-dark cycles. All experimental procedures were approved by the Medical Ethics Committee at Xiyuan Hospital, part of the China Academy of Chinese Medical Sciences (Approval No.: 2023XLC027-3; Laboratory Animal Use License No.: SYXK (Jing) 2023-0053).

#### 2.1.3 Reagents

Interleukin-6 (IL-6, batch number CK6E19B) and C-reactive protein (CRP, batch number CK5E90B) ELISA detection kits were procured from Cohesion Biosciences. The progesterone (P, batch number Apr2024) and estradiol (E2, batch number Apr2024) ELISA detection kits were obtained from Irons Biotechnology Co., Ltd. The Swiss Giemsa stain (MO62) was obtained from Shanghai Gefan Biotechnology Co., Ltd. Antibodies for phospho-Akt (Ser473) (9271S), Akt (9272S), P-PI3K (Y199) (4228S), and PI3K (4292S) were sourced from Saixintong Biological Reagent Co., Ltd. Additionally, the primary antibody for beta-actin (10021787) was acquired from Wuhan Sanying Biotechnology Co., Ltd. The EGF primary antibody (GR3446763-2), EGFR primary antibody (1001408-36), and ERα primary antibody (1009242-39) were also obtained from the same supplier. The mouse secondary antibody (824815013220) was purchased from Huaxing Biotechnology Co., Ltd., while the rabbit secondary antibody (ATXI13161) was acquired from Abbkine Scientific Co., Ltd. RIPA lysis buffer (240003002) was obtained from Solebao Technology Co., Ltd., and the 5× loading buffer (32061019101) was sourced from Huaxing Biotechnology Co., Ltd. Additionally, Giemsa stain solution (MO62) was acquired from Shanghai Gefan Biotechnology Co., Ltd.

#### 2.1.4 Instrument

The instruments utilized in this study include a microplate reader (U.S. Porton Instrument Co., Ltd., model ELX-800), an optical microscope (Japanese Obas Corporation), an electronic balance (Ohaus Corporation, model AR2130), an electrophoresis instrument (PowerPac Basic, model 041BR308459), and a gel imager (Bio-Rad, model ChemiDoc XRS+).

### 2.2 Methods

#### 2.2.1 Cyberpharmacology studies

##### 2.2.1.1 Acquisition of active ingredient targets

The TCMSP database (https://tcmsp.91medicine.cn, 2023/12/29) was employed to screen pharmacologically active compounds. Only compounds satisfying Lipinski’s rule of five (MW ≤ 500, AlogP ≤ 5, Hdon ≤ 6, Hacc ≤ 10) were retained as potential drug candidates. Target prediction was performed by querying the SMILES notation of each compound in PubChem database (Kim S et al., 2025) (https://pubchem.ncbi.nlm.nih.gov, 2023/12/29), followed by submission to the SWISS Target Prediction database (Daina A et al., 2019) (http://swisstargetprediction.ch, 2023/12/29).

##### 2.2.1.2 Screening of AV-related targets

Disease targets were retrieved from GeneCards (Safran et al., 2010) (https://www.genecards.org, 2023/12/29) and DisGeNET databases (Piñero et al., 2020) (https://disgenet.com, 2023/12/29) using the search terms ‘Senile vaginitis’ and ‘Atrophic vaginitis’, with a minimum relevance score cutoff of 10 in GeneCards. The resulting targets were integrated and deduplicated to establish a comprehensive atrophic vaginitis-related target dataset.

##### 2.2.1.3 Construction of the protein-protein interaction (PPI) network

Target gene intersections between traditional Chinese medicine (TCM) active components and atrophic vaginitis were identified using Venny 2.1. The overlapping targets were subsequently analyzed in STRING database with the organism parameter set to “*Homo sapiens*.” Afterward, utilize Cytoscape 3.7.2 to examine the network and pinpoint crucial targets.

##### 2.2.1.4 The examination of functional enrichment in gene ontology (GO) and pathways from the kyoto encyclopedia of genes and genomes (KEGG)

The DAVID 2021 (Database for Annotation, Visualization and Integrated Discovery) platform was utilized for functional annotation clustering. DAVID integrates gene ontology (GO) terms, biological pathways (KEGG), and protein-protein interaction networks to identify statistically enriched biological themes in large gene lists. To systematically elucidate FS-related pathways, the shared FS-AV targets were subjected to functional enrichment analysis using DAVID 2021. The Functional Annotation Tool was employed to identify significantly enriched GO terms and KEGG pathways using EASE score (FDR<0.05), with results sorted by enrichment score in descending order (Dennis et al., 2003). Comprehensive GO enrichment analysis covered three ontological domains: 1) biological processes (e.g., steroid hormone biosynthesis), 2) molecular functions (e.g., estrogen receptor activity), and 3) cellular components. Statistical significance was determined by a modified Fisher’s exact test (EASE score) with Benjamini-Hochberg FDR correction (*P* < 0.05), focusing on pathways exhibiting mechanistic associations between FS’s estrogenic activity and AV therapeutic effects.

##### 2.2.1.5 Molecular docking

Theprimary bioactive components were acquired from PubChem database. The three-dimensional crystal structures of the top three target proteins were obtained from the PDB. Molecular docking conformations were visualized and analyzed with PyMOL 2.2.0 to evaluate binding interactions.

#### 2.2.2 Experimental verification

##### 2.2.2.1 Animal grouping and modeling

Adult SD rats (200–220 g) were randomly allocated into six experimental groups. Group sizes were standardized to n = 11, with the exception of the sham group and the Promestriene group (n = 10). Following 3-day acclimatization, the OVX group received bilateral ovariectomies, while sham controls underwent fat pad excision with ovarian preservation. Post-surgical prophylaxis consisted of intramuscular penicillin G (800,000 U/kg, qd × 3d). Model validation required persistent diestrus or stasis (≥5 consecutive days) confirmed by daily vaginal smear. Following 14-day vaginal treatment, animals were humanely euthanized for tissue collection. The specific groups, numbers (excluding the deceased animal) and intervention measures are detailed as follows: Sham group: incision without ovariectomy; Ovariectomy (OVX) group: bilateral OVX; *F. sophorae* extract low, medium, and high (FSE-L/M/H) dose groups (11 animals per group): 5/10/20 mg·kg^−1^·d^−1^ administered as 0.2 g/suppository vaginal suppositories alongside bilateral OVX; Promestriene (PRO) group (10 animals): 0.9 mg·kg^−1^·d^−1^ administered as vaginal cream in conjunction with bilateral OVX.

##### 2.2.2.2 Observation of vaginal epithelial cells and inflammatory cells

Vaginal smears was performed for five consecutive postoperative days using Swiss Giemsa staining, with cellular composition quantified under an optical microscope. Estrous cycle staging of the rats was monitored: during proestrus (17–21 h), follicles exhibited rapid growth, characterized by a predominance of oval nucleated epithelial cells with sporadic keratinized cells. During estrus (9–15 h), follicles matured and were subsequently eliminated, characterized by a large number of anucleated keratinized epithelial cells, with a limited quantity of nucleated epithelial cells. During metestrus (10–14 h), corpus luteum forms showed comparable proportions of irregular keratinized epithelial cells, nucleated epithelial cells, and leukocytes. During diestrus (60–70 h), the corpus luteum undergowent degeneration, characterized microscopically by prominent leukocyte infiltration with scant mucus containing nucleated epithelial cells. Model validation required persistent diestrus or stasis (≥5 consecutive days) in OVX groups concomitant with normal estrous cycling in sham-operated group, after which therapeutic interventions were initiated.

##### 2.2.2.3 Vaginal pH detection and vaginal health score

Vaginal pH was measured using precision pH test strips at days 3, 7, and 14 post-treatment. Measurements were calibrated against the manufacturer’s colorimetric reference chart to quantify pH in vaginitis-affected rats. Additionally, employ an endoscope to evaluate the vaginal health status of the rats, followed by a scoring system to quantify this health status (Simon et al., 2017).

##### 2.2.2.4 Histopathological examination

Following the final treatment, rats were deeply anesthetized by intraperitoneal injection of 3% pentobarbital sodium (0.1–0.2 mL/100 g) before abdominal aorta blood collection. Euthanasia was performed by cervical dislocation under anesthesia, followed by aseptic collection of vaginal tissues. Tissues were fixed in 4% paraformaldehyde, processed through paraffin embedding, sectioned and hematoxylin-eosin (H&E) stained. Histological evaluation was performed with an Olympus BX51 microscope, and the vaginal tissue was evaluated based on parameters such as flatness, the lower layer and the muscular layer. Quantitative analysis included: vascular congestion, stromal edema, leukocyte infiltration density and epithelial layer enumeration.

##### 2.2.2.5 Serum ELISA test

Serum samples obtained from the abdominal aorta puncture (see Methods 2.2.2.4) were used for biochemical analyses. Blood samples were centrifuged (3,000 rpm, 10 min, 4°C) to separate serum fractions. The serum supernatant was aliquoted and stored at −80°C until assayed. Serum concentrations of progesterone (P), estradiol (E2), interleukin-6 (IL-6), and C-reactive protein (CRP) were quantified using enzyme-linked immunosorbent assay (ELISA) kits following manufacturers’ protocols.

##### 2.2.2.6 Detection of protein expression of estrogen receptors ERα and epidermal growth factor receptor (EGFR) in rat vaginal tissue was conducted using immunohistochemistry

Immunohistochemical analysis was performed on paraffin-embedded vaginal tissues to evaluate target protein expression levels. Protein expression was quantified by measuring the optical density of immunoreactive areas using ImageJ software.

##### 2.2.2.7 The method of Western blotting was utilized to assess the expression levels of proteins such as Akt, phospho-Akt, PI3K, phospho-PI3K, ERα, EGF, and EGFR within the vaginal tissue of rats

Vaginal tissues were minced into small pieces on a chilled platform to preserve protein integrity. Tissues were homogenized in ice-cold RIPA buffer at 4°C. Homogenates were incubated on ice for 30 min. This process was essential as it helped to maintain the stability of the samples and slowed down enzymatic reactions that could potentially alter the results. Subsequently, homogenates were centrifuged (12,000 rpm, 10 min) at 4°C. The supernatant was collected for protein quantification using a BCA assay kit. Preparing an SDS-PAGE gel and denaturing the protein samples with a boiling water bath. Equal amounts of protein were electrophoresed. Proteins were transferred to PVDF membranes, blocked with non-fat milk, and incubated with primary antibodies (1:10,000 dilution, 4°C). Membranes were washed four times with TBST before incubation with secondary antibodies (1:5,000 dilution, 45 min). After TBST washes six times, signals were detected using ECL luminescent solution and captured with a imaging system. Ultimately, band intensities were quantified using Gel-Pro-Analyzer software. Quantitative data analysis was performed by calculating the grayscale intensity ratio between the target protein and the internal reference protein β-actin, thereby indicating the relative expression levels of the target protein.

##### 2.2.2.8 Statistical methods

Statistical analyses were conducted using SPSS 14.0. Intergroup comparisons were performed by one-way ANOVA. When the assumptions of normal distribution and homogeneity of variances were not met, an independent nonparametric test was used. *P* < 0.05 and *P* < 0.01 were considered statistically significant.

## 3 Results

### 3.1 Results of a network pharmacology study of FS for the treatment of AV

#### 3.1.1 Identification of FS drug targets

The phytochemical profile of “*Fructus sophorae*” was retrieved from TCMSP database, with 12 bioactive compounds selected according to Lipinski’s rule of five (MW ≤ 500, AlogP ≤ 5, Hdon ≤ 6, Hacc ≤ 10). The Swiss Target Prediction database was employed to identify the target points of the candidate compounds. After removing redundant entries, 237 non-redundant targets were obtained through target union analysis.

#### 3.1.2 Identification of disease targets

Disease targets were systematically retrieved from GeneCards database using the search terms ‘Senile vaginitis’ and ‘Atrophic vaginitis’ with a minimum relevance score cutoff of 10. Additional targets were acquired from DisGeNET database. After integration and deduplication across datasets, 1777 unique targets were identified as being associated with AV pathogenesis.

#### 3.1.3 Identification of therapeutic targets for FS against AV and PPI network construction

Target intersection analysis between FS active components and AV-related genes was performed, identifying 100 shared targets (Figure 2A). The overlapping targets were analyzed in String 11.1 database (*Homo sapiens*; Medium confidence score >0.4) to construct FS-AV therapeutic protein-protein interaction (PPI) network. Network visualization and topological analysis were conducted using Cytoscape 3.10.0 software. Isolated nodes were excluded, and hub nodes (degree >5) were selected for further analysis. The final network comprised 55 nodes and 583 edges (Figure 2B). In this context, a higher degree value correlated with a larger node size, a darker color, an increased graphic area and a thicker connection, indicating a stronger correlation. The top four hub genes (EGFR, AKT1, TNF, ESR1) based on degree centrality represent potential core therapeutic targets of FS for AV treatment.

**FIGURE 1:**
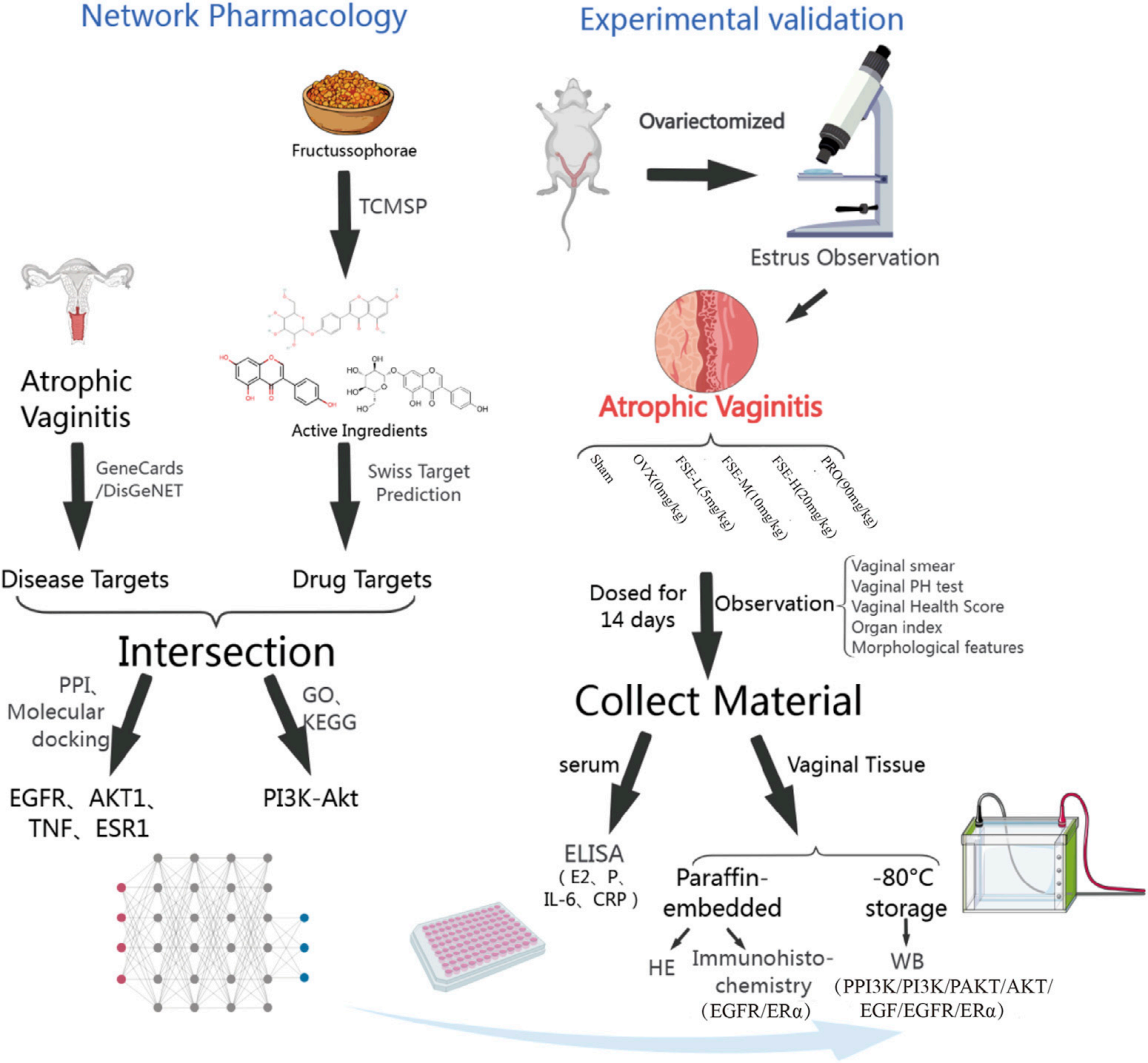
Experimental flow chart (Created with MedPeer (www.medpeer.cn)).

#### 3.1.4 GO and KEGG pathway analysis

**FIGURE 2:**
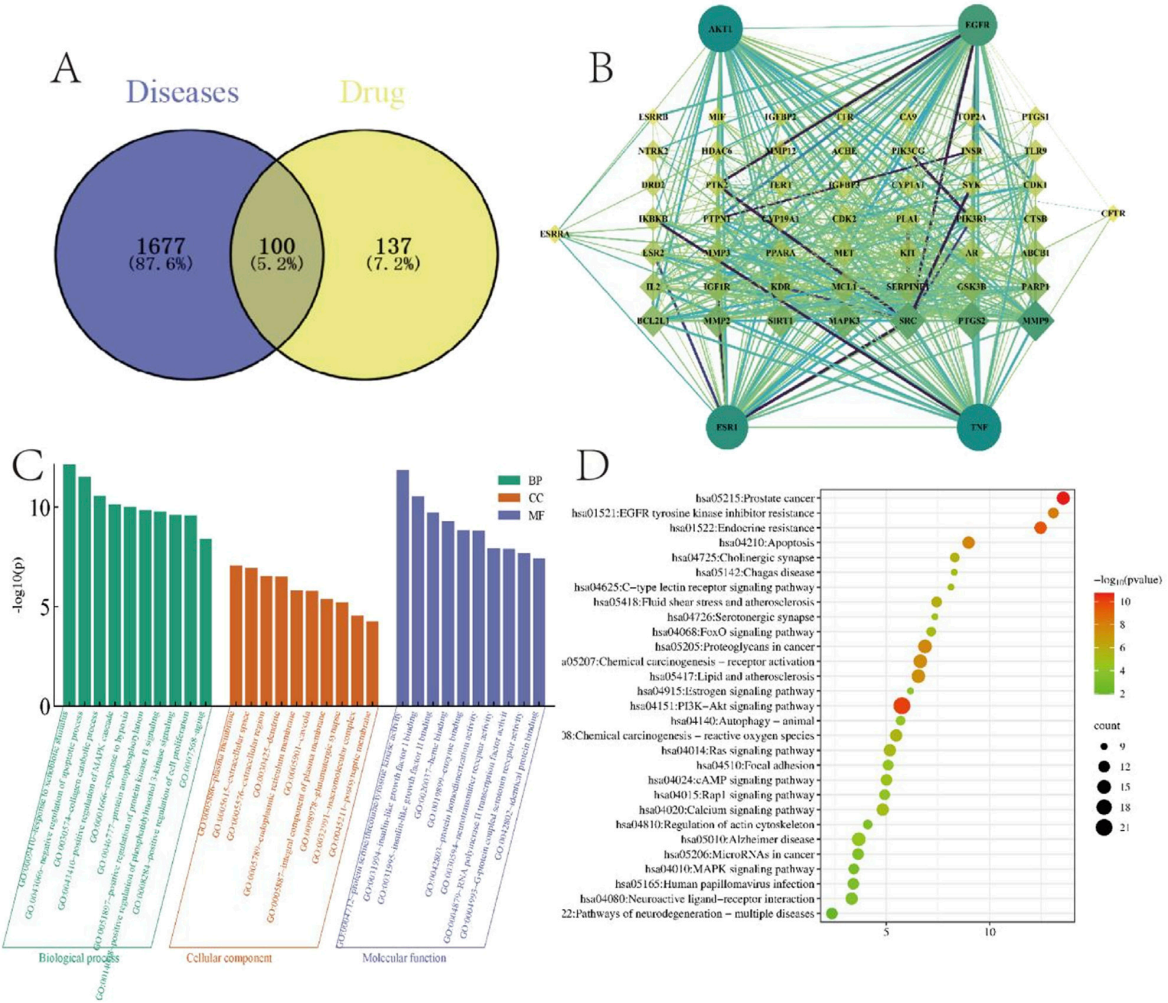
Network pharmacological target screening and enrichment. **(A)** Venn diagram of the *Fructus sophorae* target and the atrophic vaginitis target. **(B)** Key target PPI network of *Fructus sophorae* for the treatment of atrophic vaginitis. **(C)** GO function enrichment analysis. **(D)** KEGG pathway enrichment analysis.

Functional enrichment analysis of 100 candidate targets was performed using DAVID database to elucidate Gene Ontology (GO) terms and KEGG pathways. A total of 746 significantly enriched GO entries (*P* < 0.05) were identified. These comprised 22 biological processes (BP), 70 cellular components (CC), and 111 molecular functions (MF), demonstrating multifaceted pharmacological mechanisms. This thorough examination underlined the rich diversity of biological roles and functional properties associated with the potential targets, offering valuable insights for further research. For visual analysis, the top 10 BP, CC, and MF entries were chosen (Figure 2C). Top-ranked BP included apoptosis regulation and extracellular matrix remodeling (e.g., collagen catabolism). KEGG analysis revealed 130 significant pathways (*P* < 0.05), with the top 30 shown in Figure 2D based on enrichment factor. Core pathways included: PI3K-Akt signaling, VEGF signaling, Ras signaling, FoxO signaling, calcium signaling C-type lectin receptor signaling and cAMP signaling. Notably, PI3K/Akt signaling pathway contained the most targets (n = 22), suggesting its central role in FS’s therapeutic effects.

#### 3.1.5 Verification of the binding potential between soph and its therapeutic targets was conducted using molecular docking techniques

**FIGURE 3:**
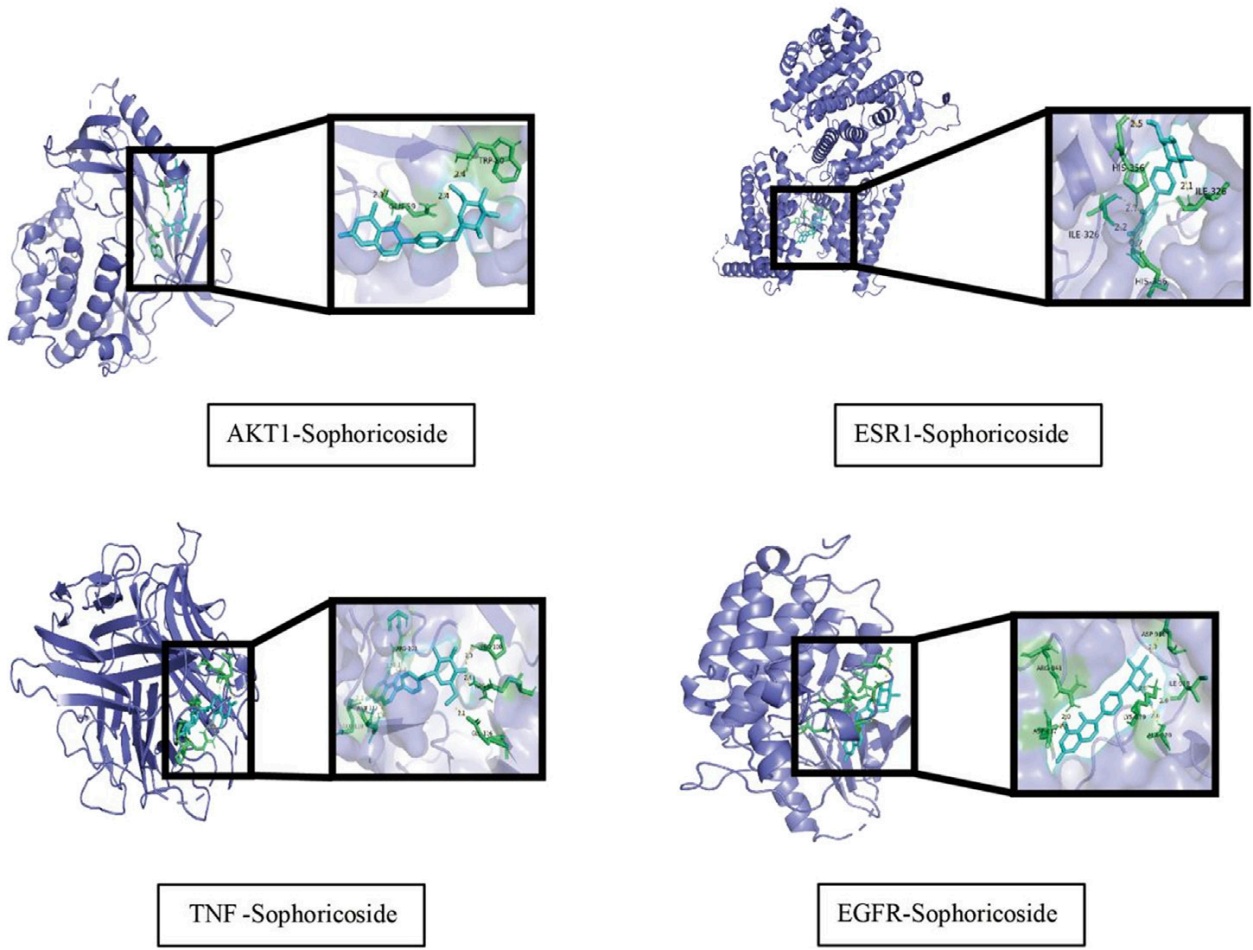
Diagram of molecular docking patterns of key components and target proteins of *Fructus sophorae* for atrophic vaginitis treatment.

Molecular docking simulations of sophoricoside (Soph, the predominant component in FSE) with core targets (EGFR, AKT1, TNF, ESR1) were conducted using AutoDock Vina 1.2.3. Protein structures were obtained from the PDB (EGFR: 5ug9, AKT1: 3o96, TNF: 2e7a, ESR1: 6vpf), preprocessed via water removal and nonpolar hydrogen deletion. The ligand (Soph) and receptors were converted to PDBQT format, with docking grids centered on catalytic sites (e.g., EGFR: center_x = 15.2, center_y = −8.5, center_z = 52.1; size = 25 × 25 × 25 Å^3^). Docking parameters included an exhaustiveness of 20 and maximum binding modes of 10. Soph exhibited strong binding affinities: TNFTNF (−9.8 kcal·mol^-1^), AKT1 (−8.2 kcal·mol^-1^), ESR1 (−8.2 kcal·mol^-1^), and EGFR (−7.9 kcal·mol^-1^), suggesting spontaneous complex formation (Table 1). All docked poses showed RMSD < 2.5 Å (TNF: 0.865 Å; ESR1: 0.995 Å; EGFR: 1.398 Å; AKT1: 2.063 Å), validating stable ligand conformations. Detailed methods are available in Supplementary Information. Binding interactions are depicted in Figure 3.

**TABLE 1:** Binding energy of key targets and core components of *Fructus sophorae* for the treatment of atrophic vaginitis.

Active ingredient	Target proteins	Protein no.	Binding Energy/(kcal/mol)	Root mean square deviation
Sophoricoside	AKT1	3o96	−8.2	2.063
	ESR1	6vpf	−8.2	0.995
	TNF	2e7a	−9.8	0.865
	EGFR	5ug9	−7.9	1.398

### 3.2 Results of pharmacodynamic experiments

#### 3.2.1 Effect of FSE on uterine and vaginal tissues of ovariectomised Rats

**FIGURE 4:**
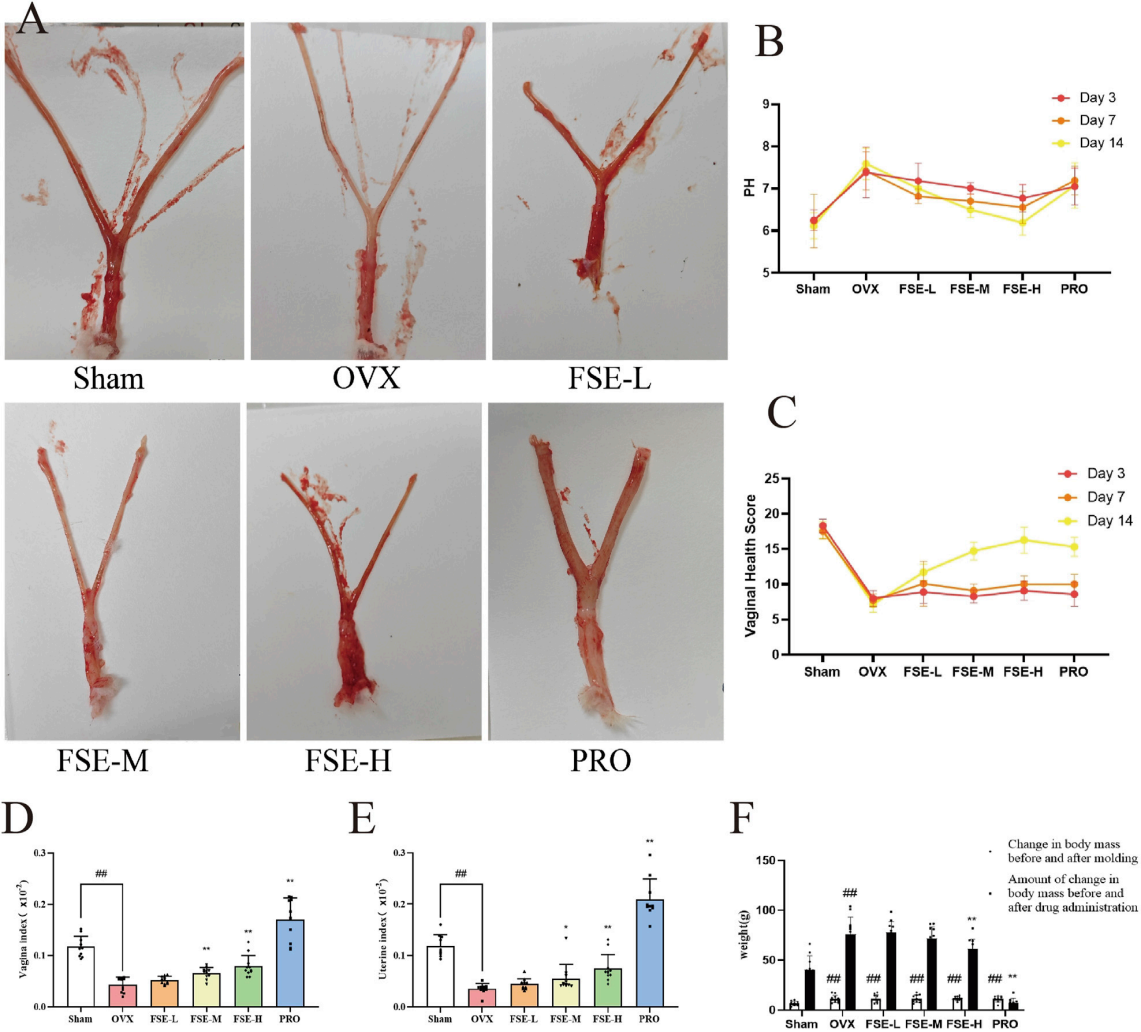
Effect of FSE on Uterine and Vaginal Tissues of Ovariectomised Rats. **(A)** Vaginal and uterine morphological characteristics. **(B)** Intravaginal pH. **(C)** Vaginal Health Score. **(D)** Vagina index. **(E)** Uterus index. **(F)** Weight of rats. Data are the mean ± SD (n ≥ 10). ^#^ *P* < 0.05 and ^##^ *P* < 0.01 compared to the Sham group.^*^ *P* < 0.05 and ^**^ *P* < 0.01 compared to the OVX group.

Post-OVX rats developed characteristic atrophic changes: 1) well-demarcated vaginal/uterine atrophy, 2) reduced vascularization, and 3) cervical softening (Figure 4A), confirming successful AV model induction, and accelerated weight gain in rats after surgery (Figure 4F). Compared with the sham operation (Sham) group, OVX rats showed significantly elevated vaginal pH (*P* < 0.05) and reduced vaginal health scores. The drug administration group significantly ameliorated these atrophic parameters (Figures 4B,C). Additionally, OVX rats exhibited significant vaginal atrophy and lower vaginal index than the Sham group (Figure 4D). Therapeutic intervention with FSE and PRO restored vaginal weight (*P* < 0.05). Furthermore, a significant reduction in the uterine coefficient was observed in the OVX group compared to the Sham group (*P* < 0.01). Notably, the FSE-M/H and PRO group showed a notable reversal of uterine atrophy compared to the OVX group, resulting in a considerable increase in the uterine coefficient (*P* < 0.01), as shown in Figure 4E. Collectively, these data demonstrate that OVX-induced hypoestrogenism causes vaginal alkalization and vaginal and uterine atrophy, which are significantly attenuated by FSE therapy.

#### 3.2.2 Effect of FSE on microorganisms in the rat vagina

**FIGURE 5:**
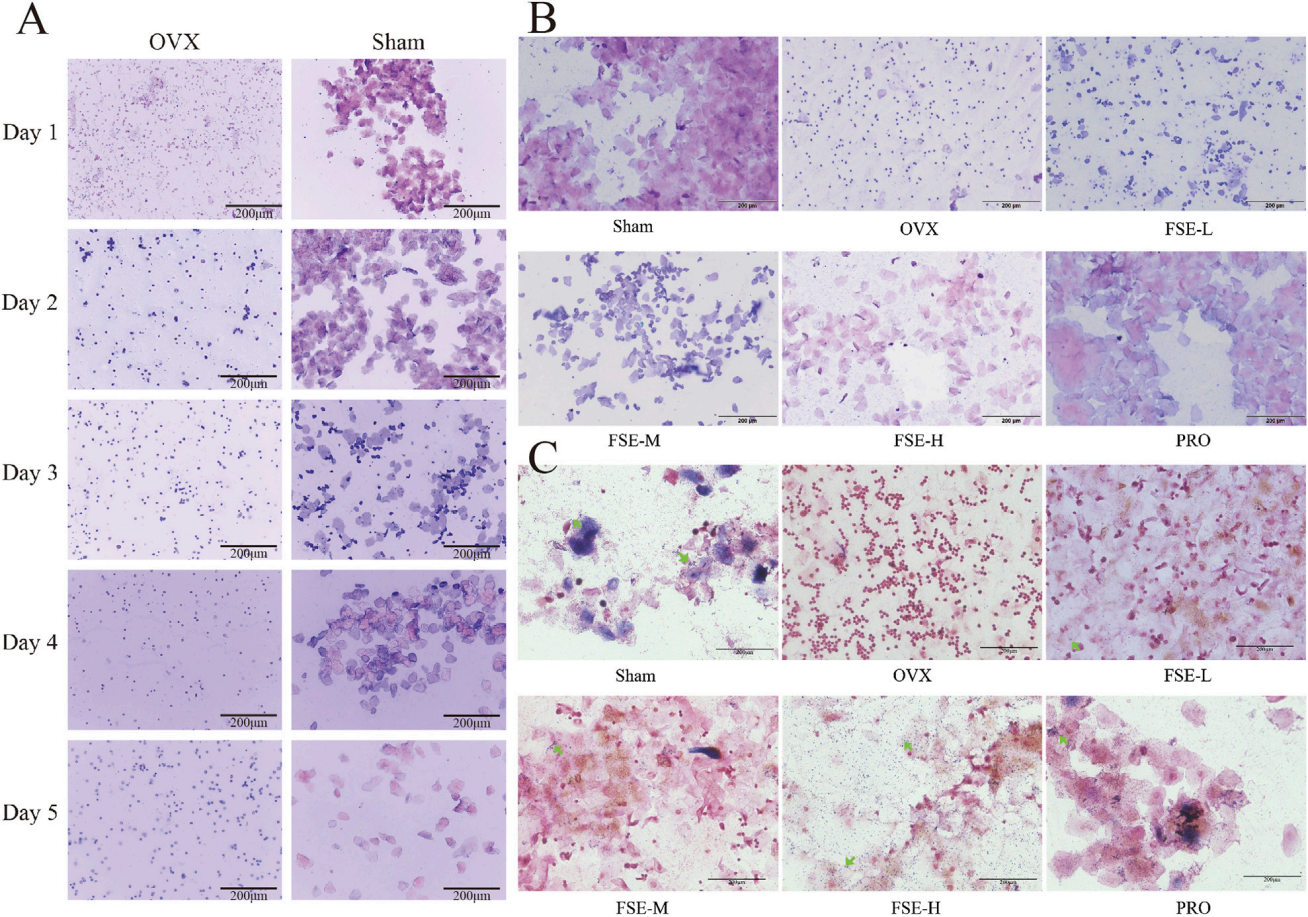
Staining results of rat vaginal smear. **(A)** Swiss Giemsa-stained vaginal smears collected daily for five consecutive days post-surgery. **(B)** Swiss Giemsa staining of vaginal smears on the 14th day of drug administration. **(C)** Gram staining of vaginal smears on the 14th day of dosing. (40×) (n ≥ 10).

Postoperative vaginal smears in OVX rats showed interestrus for five consecutive days, characterized by a significant presence of leukocytes and a scarcity of squamous epithelial cells. In contrast, the sham group maintained alterations in the estrous cycle (Figure 5A), validating model efficacy and permitting therapeutic interventions.

Vaginal mucosal atrophy predisposes to pathogenic bacterial overgrowth, significantly increasing postmenopausal women’s susceptibility to recurrent vaginitis. As shown in Figures 5B,C, Gram/Swiss Giemsa-stained vaginal smears from the Sham group exhibited dense rod-shaped lactobacilli and polygonal squamous exfoliated cells, confirming a normal vaginal microecology. Conversely, smears from the OVX group revealed reduced lactobacilli, elevated leukocyte levels and the presence of impurity-associated staining patterns in the vaginal secretions. FSE/PRO treatment restored lactobacilli counts, along with a prevalence of keratinized or non-keratinized epithelial cells, and reduced inflammatory cells infiltration. The increase in vaginal lactobacilli was associated with vaginal pH normalization in the FSE and PRO groups compared to the OVX group (Figure 5C).

#### 3.2.3 Effect of FSE on vaginal morphology in rats

**FIGURE 6:**
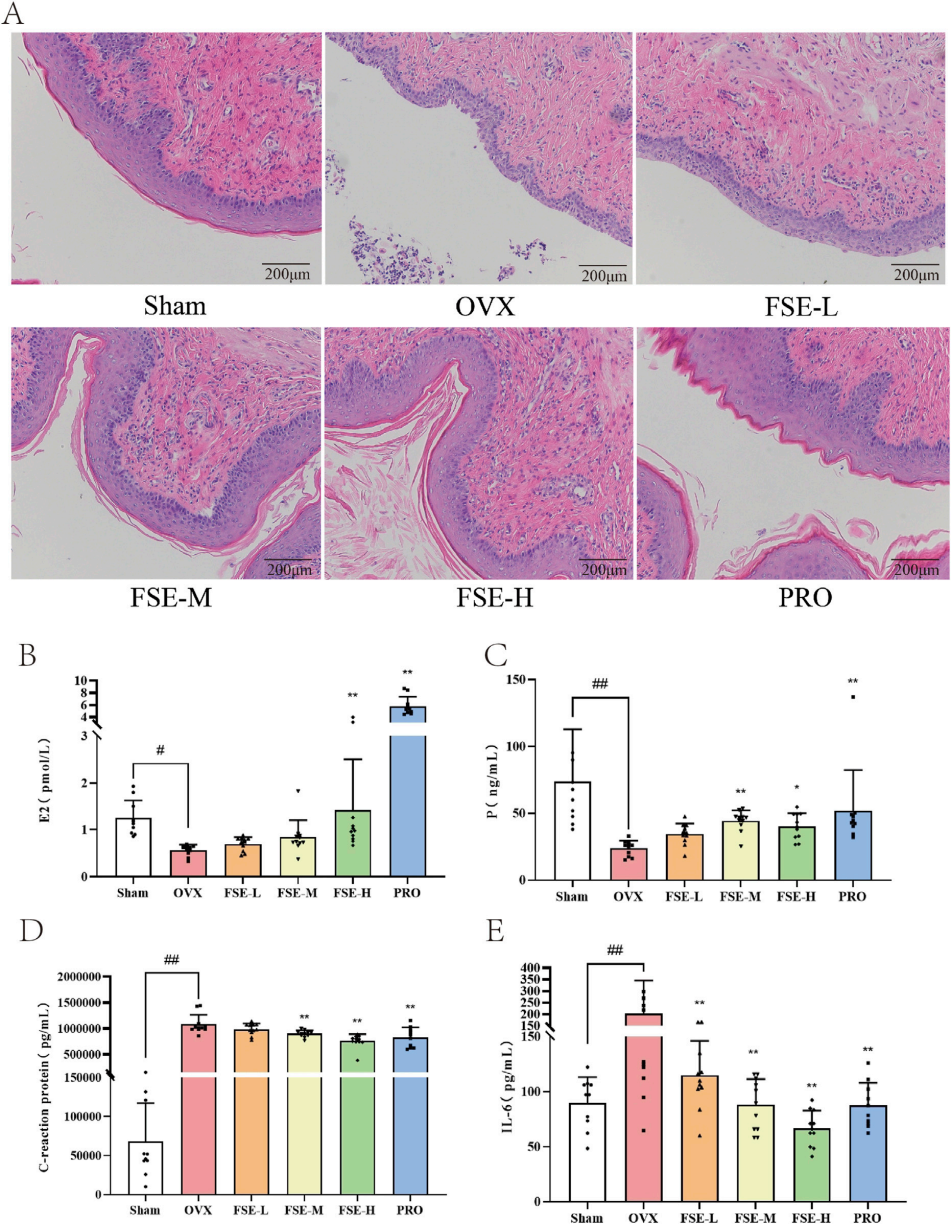
Effects of FSE on histomorphology and serum estrogen and inflammatory factors in ovariectomized rats were examined. **(A)** HE staining analysis (20×). **(B)** E2 levels. **(C)** P levels. **(D)** CRP levels. **(E)** IL-6 levels. Data are the mean ± SD (n ≥ 10). ^#^ *P* < 0.05 and ^##^ *P* < 0.01 compared to the Sham group.^*^ *P* < 0.05 and ^**^ *P* < 0.01 compared to the OVX group.

Morphological alterations in the vagina were identified through H&E staining. As shown in Figure 5A, the vaginal epithelial structure in the Sham group appeared a well-preserved and thickened vaginal epithelium, exhibiting a complex squamous epithelium characterized by keratinization upon microscopic evaluation. The lamina propria exhibited dense vascularization within robust connective tissue. In contrast, the OVX group exhibited severe epithelial atrophy (2-3 cell layers), with complete keratinization loss. This group exhibited either localized or widespread defects in the mucosal layer, accompanied by significant infiltration of inflammatory cells. Compared to the OVX group, the vaginal basal epithelial cells from the FSE and PRO groups demonstrated enhanced proliferative activity, along with a significantly thickened, well-organized stratified squamous epithelium. These findings indicate that FSR exhibits comparable therapeutic efficacy to PRO, significantly increasing vaginal wall thickness and enhancing epithelial integrity (Figure 6A).

#### 3.2.4 Effect of FSE on hormone levels and inflammatory factor levels in ovariectomised rats

Serum concentrations of serum estrogen (E2), progesterone (P), interleukin 6 (IL-6), and C-reactive protein (CRP) were quantified to evaluate FSE’s hormone-modulating and anti-inflammatory effects in OVX rats. As shown in Figures 6B,C, the serum concentrations of E2 and P in the ovariectomized (OVX) group were significantly lower than those observed in the Sham (*P* < 0.05), FSE-M/H, and PRO groups (*P* < 0.01). The hormonal levels alterations observed following ovariectomy induction recapitulate the characteristic endocrine milieu of natural postmenopausal status in women. Compared to the OVX group, both FSE and PRO administration resulted in statistically significant elevations in estrogen (E2) levels (*P* < 0.05). Furthermore, the OVX group manifested significantly higher levels of the inflammatory markers CRP and IL-6 compared with the Sham group (*P* < 0.01). Conversely, the treatment groups demonstrated statistically significant attenuation of these inflammatory factors compared to the OVX group (*P* < 0.01), as shown in Figures 6D,E. These data confirmed OVX-induced systemic inflammation and established FSE’s dual estrogenic and anti-inflammatory efficacy comparable to standard hormone therapy.

### 3.3 Effect of FS on target proteins

#### 3.3.1 Immunohistochemical method was employed to assess the protein expression levels of ERα and EGFR in the vaginal tissue of rats

**FIGURE 7:**
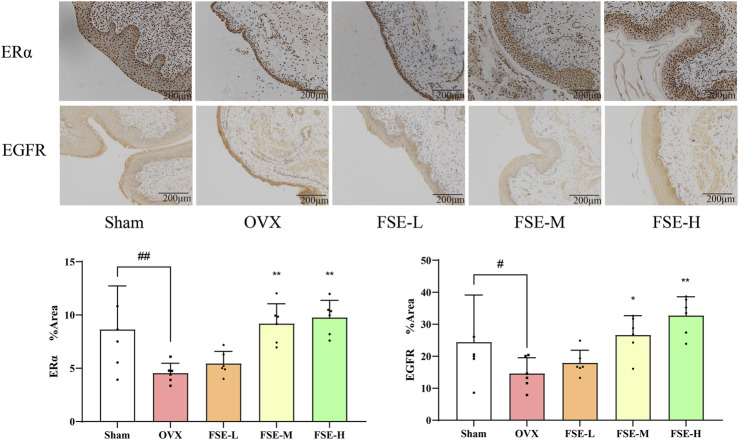
Effect of *Fructus sophorae* extract on the expression of ERα and EGFR proteins in vaginal tissues of bilateral ovariectomized rats (immunohistochemical staining, 20×, n = 6).

Immunohistochemical analysis revealed significantly diminished ERα and EGFR protein expression in vaginal tissues of the OVX group compared to the Sham group (*P* < 0.05 or *P* < 0.01). Conversely, the FSE-M/H group showed a significant enhancement in the protein expression of these proteinscompared to the OVX group (*P* < 0.05 or *P* < 0.01), as shown in Figure 7.

#### 3.3.2 The Western blot method was employed to assess the protein expression levels of ERα, EGF, EGFR, p-AKT, AKT, p-PI3K, PI3K in the vaginal tissue of rats

**FIGURE 8:**
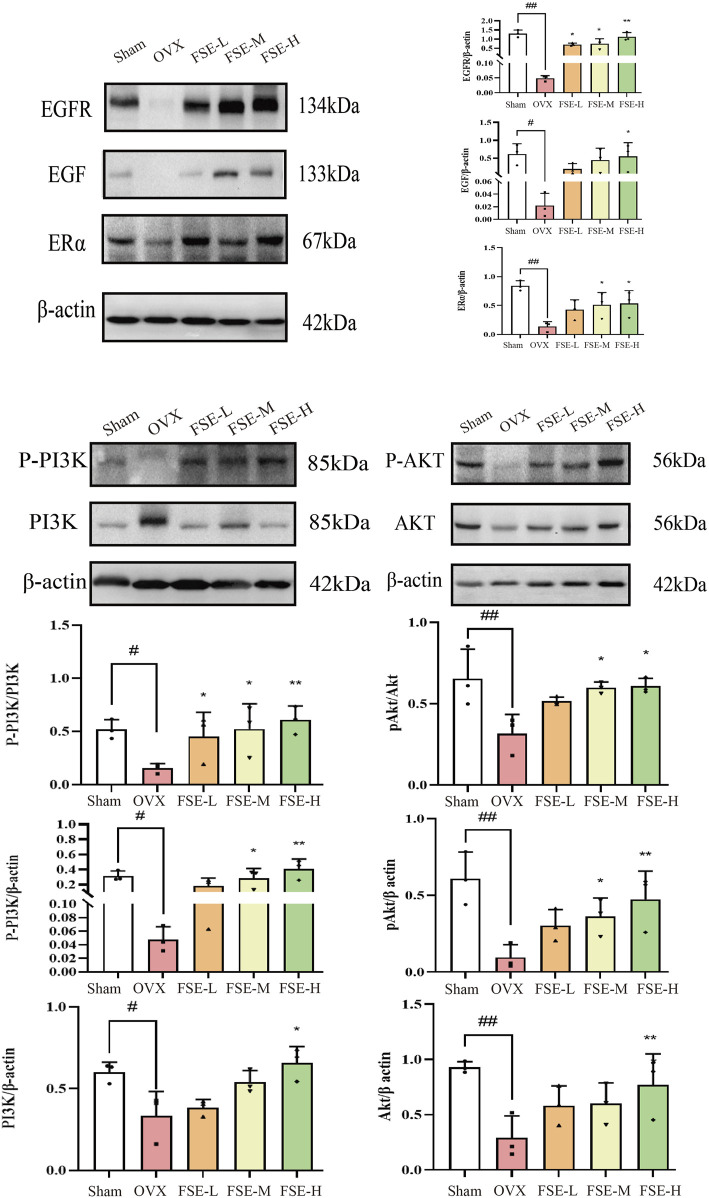
Effect of *Fructus sophorae* extract on the expression of ERα, EGF, EGFR, P-PI3K, PI3K, PAKT, and AKT proteins in vaginal tissues of bilaterally ovariectomized rats (n = 3).

Quantitative proteomic analysis revealed marked downregulation of ERα, EGF, EGFR, p-AKT, AKT, p-PI3K, and PI3K in vaginal tissues of OVX rats compared to the Sham group (*P* < 0.05 or *P* < 0.01). Conversely, Compared to the OVX group, the expression levels of vaginal tissue proteins in rats of FSE administration group were significantly increased (*P* < 0.05 or *P* < 0.01), as shown in Figure 8.

## 4 Discussion

As a widely recognized treatmen for atrophic vaginitis (AV), estrogen replacement therapy (ERT) demonstrates clinical efficacy in postmenopausal women through dual mechanisms: normalizing estrogen levels and enhancing *Lactobacillus* proliferation, thereby restoring vaginal mucosal integrity and function (Krause et al., 2009). ERT delivery systems include systemic (oral/transdermal) and topical (vaginal creams/tablets) formulations. Notwithstanding its efficacy, long-term estrogen-progestin therapy correlates with increased breast cancer incidence and treatment-limiting adverse effects (e.g., mastalgia, breast swelling), contraindicated in patients with prior hormone-sensitive malignancies or documented thromboembolic disorders (Bhupathiraju et al., 2019). Topically administered low-dose estrogen preparations achieve symptomatic relief while minimizing systemic absorption (Crandall et al., 2018), establishing them as the formulation of choice due to their localized action profile and favorable safety parameters. Although clinically beneficial, persistent concerns about prolonged estrogen exposure and its oncogenic potential result in suboptimal treatment adherence, potentially worsening AV pathology (Kingsberg et al., 2019). These limitations highlight the urgent need for investigating phytoestrogens (e.g., Sophoricoside) as viable therapeutic alternatives, offering the potential to circumvent synthetic hormone-related risks.

To elucidate the molecular mechanism underlying FS’s therapeutic effects, we performed molecular docking validation. Given that Sophoricoside (Soph) accounts for the major bioactive component of FSE, it was selected as for this molecular docking analysis. Molecular docking results were evaluated based on the calculated binding free energy (ΔG) (Huang et al., 2021). A reduced binding energy signals that a smaller amount of energy was necessary for the drug molecule to bind to the target, indicating an increased probability and stability of that binding interaction (Murugan et al., 2020; Kadela-Tomanek et al., 2021). Integrative analysis combining molecular docking with *in vitro* and *in vivo* experiments demonstrated FS’s therapeutic potential against OVX-induced AV via modulation of ERα, EGF, EGFR, P-PI3K, PI3K, PAKT, and AKT signaling pathways. And molecular docking experiments need to be corroborated by *ex vivo* and *in vivo* experiments (Zhou et al., 2020).

Estrogen deficiency disrupted vaginal homeostasis through diminished *Lactobacillus* dominance and impaired lactic acid production, resulting in elevated vaginal pH (>5.5) (Calleja-Agius and Brincat, 2015) that facilitates pathogenic overgrowth, as demonstrated in our OVX rat model. FSE treatment restored vaginal pH and Gram-positive bacilli predominance, with concomitant serum E2 elevation (OVX: 0.57 ± 0.034 vs. FSE-H: 1.41 ± 0.33 pg/mL; *P* < 0.01), indicating intrinsic estrogenic activity. Histopathological assessment confirmed FSE-H mediated reversal of OVX-induced epithelial atrophy and inflammatory infiltration, validating its mucosa-protective efficacy (Binder et al., 2019). Notably, whereas conjugated estrogens (PRO) provoked uterine hyperplasia (uterine coefficient: PRO: 0.21 ± 0.01 vs. FSE-H: 0.07 ± 0.01; *P* < 0.05), FSE exerted comparable therapeutic effects without hyperproliferative risks, potentially via selective estrogen receptor modulation and anti-inflammatory pathways (e.g., TNF/NF-κB inhibition). This pleiotropic therapeutic profile established FSE as a viable AV treatment option, simultaneously addressing microbial dysbiosis, epithelial integrity, and hormonal function.

ESR1, also known as ERα, is a ligand-activated transcription factor critical for estrogen-dependent gene regulation. It plays a pivotal role in the pathogenesis of atrophic vaginitis (AV), particularly in postmenopausal women. The postmenopausal decline in estrogen levels represents a key determinant of vaginal epithelial homeostasis. This hormonal shift constitutes a pivotal physiological change, inducing various systemic alterations that may compromise the homeostasis of the vaginal microenvironment. Accumulated evidence highlights ERα′s critical involvement in preserving vaginal epithelial morphology and physiological function. Consequently, maintaining optimal estrogen levels and preserved ERα activity are essential for preserving vaginal tissue integrity and function in post-menopausal women (Kim et al., 2023). Estrogen’s physiological actions are principally mediated via ERα ligand binding and subsequent activation. The receptor mediates crucial transcriptional regulation through interaction with cis-regulatory sequences termed estrogen response elements (EREs), located within the promoters of its target genes Our experimental data revealed significantly downregulated ERα expression in OVX rats. These results corroborated the well-documented association between estrogen receptor signaling and AV pathogenesis. Moreover, our data implied that FSE confers mucosal protection against AV through estrogen receptor-mediated mechanisms. Notably, estrogens can elicit rapid, non-genomic signaling cascades through membrane-associated receptors. These membrane-initiated events activate key signaling pathways including MAP and PI3K/AKT cascades. These mechanisms highlight the multifaceted role of estrogens in regulating various biological processes beyond traditional genomic actions, showcasing their importance in signaling and potential therapeutic applications in conditions related to estrogen receptor activity (Chen et al., 2015).

Quantitative analysis revealed significant downregulation of PI3K/AKT pathway components in the OVX group, demonstrating estrogen-dependent regulation of this pathway and suggesting FSE’s therapeutic potential to normalize pathway activity. Interestingly, the estrogen pathway may also influence growth factor receptor signaling, including that of EGFR and IGF-IR (Georgakilas et al., 2017), with EGF capable of inducing phosphorylation of ERα at Ser118 and stimulating its transcriptional activity (Li et al., 2023). The EGFR signaling axis emerges as a key modulator of AV pathogenesis. The postmenopausal estrogen deficiency induces profound alterations in vaginal epithelial homeostasis, characterized by glycogen depletion and microbial dysbiosis, thereby predisposing to infectious complications and aggravating atrophic manifestations. Pharmacological activation of EGFR signaling represents a potential therapeutic strategy to counteract these changes through epithelial proliferation and tissue remodeling, thereby preserving vaginal mucosal integrity (Hall et al., 2001). Our experimental data revealed significant downregulation of EGF/EGFR expression in the OVX group, that was subsequently reversed by FSE treatment, establishing a pathophysiological link between this signaling axis and AV progression. These findings supported FSE’s therapeutic efficacy in modulating the EGF/EGFR pathway to ameliorate AV-associated epithelial dysfunction.

This multidisciplinary investigation (network pharmacology, molecular docking and preclinical validation) established ERα, EGFR, and PI3K/AKT signaling as key mechanistic targets for FSE’s therapeutic effects in AV, demonstrated by restoration of ERα, EGFR and p-AKT expression in OVX models. While these findings illuminated FSE’s multi-target mechanism (e.g., estrogenic activity mediated through ERα and epithelial facilitated via the EGFR/PI3K pathway), translating these insights into clinical applications necessitates caution: (1) While the OVX rat model represented the gold standard for postmenopausal AV research, it exhibited limitations in fully recapitulating human vaginal microbiome composition and functional dynamics; (2) Dose-response relationships and long-term safety of FSE-derived phytoestrogens (e.g., Sophoricoside) remained to be established in human trials; (3) Synergistic effects between FSE constituents warranted further pharmacokinetic characterization to optimize formulations. These findings supported FSE’s development as a microbiome-sparing adjuvant to low-dose vaginal estrogen, especially for breast cancer survivors with contraindications to systemic hormone therapy. Future research should explore the relationship between vaginal pH normalization and *Lactobacillus* recovery in clinical cohorts, while integrating these findings with the observed EGFR/PI3K pathway modulation by FSE in the current study, thereby establishing a translational bridge from preclinical mechanisms to patient-centered outcomes.

## Data Availability

The datasets presented in this study can be found in online repositories. The names of the repository/repositories and accession number(s) can be found in the article/Supplementary Material.

## References

[B1] Abdallah H. M. Al-Abd A. M. Asaad G. F. Abdel-Naim A. B. El-halawany A. M. (2014). Isolation of antiosteoporotic compounds from seeds of Sophora japonica. PLoS One 9 (6), e98559. 10.1371/journal.pone.0098559 24892557 PMC4043785

[B2] Benini V. Ruffolo A. Casiragi A. Degliuomini R. S. Frigerio M. Braga A. (2022). New innovations for the treatment of vulvovaginal atrophy: an up-to-date review. Medicina 58 (6), 770. 10.3390/medicina58060770 35744033 PMC9230595

[B3] Bhupathiraju S. N. Grodstein F. Stampfer M. J. Willett W. C. Crandall C. J. Shifren J. L. (2019). Vaginal estrogen use and chronic disease risk in the Nurses’ Health Study. Menopause 26 (6), 603–610. 10.1097/gme.0000000000001284 PMC6538478 30562320

[B4] Binder R. L. Freedman M. A. Sharma K. B. Farage M. A. Wang Y. Combs C. (2019). Histological and gene expression analysis of the effects of menopause status and hormone therapy on the vaginal introitus and labia majora. J. Clin. Med. Res. 11 (11), 745–759. 10.14740/jocmr4006 31803317 PMC6879024

[B5] Calleja-Agius J. Brincat M. P. (2015). The urogenital system and the menopause. Climacteric 18 (Suppl. 1), 18–22. 10.3109/13697137.2015.1078206 26366796

[B6] Chakuleska L. Shkondrov A. Popov G. Zlateva-Panayotova N. Petrova R. Atanasova M. (2022). Beneficial effects of the fructus Sophorae extract on experimentally induced osteoporosis in New Zealand white rabbits. Acta Pharm. 72 (2), 289–302. 10.2478/acph-2022-0012 36651509

[B7] Chen Z. Zhang Z. Zhang H. Xie B. (2015). Analysis of the oxidative stress status in nonspecific vaginitis and its role in vaginal epithelial cells apoptosis. Biomed. Res. Int. 2015, 795656. 10.1155/2015/795656 26558281 PMC4628999

[B8] Chua Y. Limpaphaayom K. K. Cheng B. Ho C. M. Sumapradja K. Altomare C. (2017). Genitourinary syndrome of menopause in five Asian countries: results from the Pan-Asian REVIVE survey. Climacteric 20 (4), 367–373. 10.1080/13697137.2017.1315091 28453308

[B9] Cox S. Nasseri R. Rubin R. S. Santiago-Lastra Y. (2023). Genitourinary syndrome of menopause. Med. Clin. North Am. 107 (2), 357–369. 10.1016/j.mcna.2022.10.017 36759102

[B10] Crandall C. J. Hovey K. M. Andrews C. A. Chlebowski R. T. Stefanick M. L. Lane D. S. (2018). Breast cancer, endometrial cancer, and cardiovascular events in participants who used vaginal estrogen in the Women's Health Initiative Observational Study. Menopause 25 (1), 11–20. 10.1097/GME.0000000000000956 28816933 PMC5734988

[B11] Daina A. Michielin O. Zoete V. (2019). SwissTargetPrediction: updated data and new features for efficient prediction of protein targets of small molecules. Nucleic Acids Res. 47 (W1), W357–W364. 10.1093/nar/gkz382 31106366 PMC6602486

[B12] DennisG.ShermanB. T.HosackD. A.YangJ.GaoW.LaneH. C. (2003). DAVID: database for annotation, visualization, and integrated discovery. Genome Biol. 4 (5), P3. 10.1186/gb-2003-4-5-p3 12734009

[B13] dos Santos J. S. Suzan A. J. Bonafé G. A. Fernandes A. M. A. d. P. Longato G. B. Antônio M. A. (2023). Kaempferol and biomodified kaempferol from Sophora japonica extract as potential sources of anti-cancer polyphenolics against high grade glioma cell lines. Int. J. Mol. Sci. 24 (13), 10716. 10.3390/ijms241310716 37445894 PMC10341967

[B14] Georgakilas A. G. Martin O. A. Bonner W. M. (2017). p21: a two-faced genome guardian. Trends Mol. Med. 23 (4), 310–319. 10.1016/j.molmed.2017.02.001 28279624

[B15] Guo W. Huang J. Wang N. Tan H. Y. Cheung F. Chen F. (2019). Integrating network pharmacology and pharmacological evaluation for deciphering the action mechanism of herbal formula zuojin pill in suppressing hepatocellular carcinoma. Front. Pharmacol. 10, 1185. 10.3389/fphar.2019.01185 31649545 PMC6795061

[B16] Hainer B. L. Gibson M. V. (2011). Vaginitis: diagnosis and treatment. Am. Fam. Physician 83 (7), 807–815. 10.1097/SPV.0b013e3181ab4804 21524046

[B17] Hall J. M. Couse J. F. Korach K. S. (2001). The multifaceted mechanisms of estradiol and estrogen receptor signaling. J. Biol. Chem. 276 (40), 36869–36872. 10.1074/jbc.R100029200 11459850

[B18] Huang X. Pearce R. Omenn G. S. Zhang Y. (2021). Identification of 13 Guanidinobenzoyl- or Aminidinobenzoyl-containing drugs to potentially inhibit TMPRSS2 for COVID-19 treatment. Int. J. Mol. Sci. 22 (13), 7060. 10.3390/ijms22137060 34209110 PMC8269196

[B19] Jiang Z. Meng Y. Bo L. Wang C. Bian J. Deng X. (2018). Sophocarpine attenuates LPS-induced liver injury and improves survival of mice through suppressing oxidative stress, inflammation, and apoptosis. Mediat. Inflamm. 2018, 5871431. 10.1155/2018/5871431 PMC5976937 29861657

[B20] Juhász M. L. W. Korta D. Z. Mesinkovska N. A. (2021). Vaginal rejuvenation: a retrospective review of lasers and radiofrequency devices. Dermatol. Surg. 47 (4), 489–494. 10.1097/dss.0000000000002845 33165070

[B21] Kadela-Tomanek M. Jastrzębska M. Marciniec K. Chrobak E. Bębenek E. Boryczka S. (2021). Lipophilicity, pharmacokinetic properties, and molecular docking study on SARS-CoV-2 target for betulin triazole derivatives with attached 1,4-quinone. Pharmaceutics 13 (6), 781. 10.3390/pharmaceutics13060781 34071116 PMC8224687

[B22] Kim J. M. Dziobaka S. Yoon Y. E. Lee H. L. Jeong J. H. Lee I. R. (2023). OR2H2 activates CAMKKβ–AMPK–autophagy signaling axis and suppresses senescence in VK2/E6E7 cells. Pharmaceuticals 16 (9), 1221. 10.3390/ph16091221 37765029 PMC10535153

[B23] Kim S. (2016). Getting the most out of PubChem for virtual screening. Expert Opin. Drug Discov. 11 (9), 843–855. 10.1080/17460441.2016.1216967 27454129 PMC5045798

[B24] Kim S. Chen J. Cheng T. Gindulyte A. (2025). PubChem 2025 update. Nucleic Acids Res. 53 (D1), D1516–D1525. 10.1093/nar/gkae1059 39558165 PMC11701573

[B25] Kingsberg S. A. Larkin L. Krychman M. Parish S. J. Bernick B. Mirkin S. (2019). WISDOM survey: attitudes and behaviors of physicians toward vulvar and vaginal atrophy (VVA) treatment in women including those with breast cancer history. Menopause 26 (2), 124–131. 10.1097/GME.0000000000001194 30130293 PMC6365251

[B26] Krause M. Wheeler T. L. Snyder T. E. Richter H. E. (2009). Local effects of vaginally administered estrogen therapy: a review. J. Pelvic Med. Surg. 15 (3), 105–114. 10.1097/SPV.0b013e3181ab4804 22229022 PMC3252029

[B27] Lee W. Y. Lee C. Y. Kim Y. S. (2019). The methodological trends of traditional herbal medicine employing network pharmacology. Biomolecules 9 (8), 362. 10.3390/biom9080362 31412658 PMC6723118

[B28] Lester J. Pahouja G. Andersen B. Lustberg M. (2015). Atrophic vaginitis in breast cancer survivors: a difficult survivorship issue. J. Pers. Med. 5 (2), 50–66. 10.3390/jpm5020050 25815692 PMC4493485

[B29] Li D. Zhang T. Yang H. Yang W. Zhang C. Gao G. (2023). Effect of vitamin D on the proliferation and barrier of atrophic vaginal epithelial cells. Molecules 28 (18), 6605. 10.3390/molecules28186605 37764381 PMC10535479

[B30] Liu Y. Huang W. Ji S. Wang J. Luo J. Lu B. (2022). Sophora japonica flowers and their main phytochemical, rutin, regulate chemically induced murine colitis in association with targeting the NF-κB signaling pathway and gut microbiota. Food Chem. 393, 133395. 10.1016/j.foodchem.2022.133395 35691061

[B31] Mac Bride M. B. Rhodes D. J. Shuster L. T. (2010). Vulvovaginal atrophy. Mayo Clin. Proc. 85 (1), 87–94. 10.4065/mcp.2009.0413 20042564 PMC2800285

[B32] Mazalzadeh F. Hekmat K. Namjouyan F. Saki A. (2020). Effect of Trigonella foenum (fenugreek) vaginal cream on vaginal atrophy in postmenopausal women. J. Fam. Med. Prim. Care 9 (6), 2714–2719. 10.4103/jfmpc.jfmpc_1220_19 PMC7491783 32984113

[B33] Murugan N. A. Muvva C. Jeyarajpandian C. Jeyakanthan J. Subramanian V. (2020). Performance of force-field- and machine learning-based scoring functions in ranking MAO-B protein–inhibitor complexes in relevance to developing Parkinson’s therapeutics. Int. J. Mol. Sci. 21 (20), 7648. 10.3390/ijms21207648 33081086 PMC7589968

[B34] Patti A. M. Al-Rasadi K. Giglio R. V. Nikolic D. Mannina C. Castellino G. (2018). Natural approaches in metabolic syndrome management. Arch. Med. Sci. 14 (2), 422–441. 10.5114/aoms.2017.68717 29593818 PMC5868676

[B35] Piñero J. Ramírez-Anguita J. M. Saüch-Pitarch J. Ronzano F. Centeno E. Sanz F. (2020). The DisGeNET knowledge platform for disease genomics: 2019 update. Nucleic Acids Res. 48 (D1), D845–D855. 10.1093/nar/gkz1021 31680165 PMC7145631

[B36] Safran M. Dalah I. Alexander J. Rosen N. Iny Stein T. Shmoish M. (2010). GeneCards Version 3: the human gene integrator. Database 2010, baq020. 10.1093/database/baq020 20689021 PMC2938269

[B37] Simon J. A. Archer D. F. Kagan R. Bernick B. Graham S. Constantine G. D. (2017). Visual improvements in vaginal mucosa correlate with symptoms of VVA: data from a double-blind, placebo-controlled trial. Menopause 24 (9), 1003–1010. 10.1097/GME.0000000000000880 28419068 PMC5571882

[B38] Sun G. Huo J. Pan W. (2019). Analysis of chemical components of Sophora japonica medicinal materials based on UPLC-Q-TOF/MS technology. Chin. Herb. Med. 50 (16), 3774–3783. 10.7501/j.issn.0253-2670.2019.16.001

[B39] Ulhe S. C. Acharya N. Vats A. K S. Mohanty P. K. (2024). Tacrolimus concentration/dose ratio: a Tool for guiding tacrolimus dosage post-renal transplantation. Cureus 16 (2), e53421. 10.7759/cureus.53421 38435193 PMC10908598

[B40] Wu Z. Ma H. Liu Z. Zheng L. Yu Z. Cao S. (2022). wSDTNBI: a novel network-based inference method for virtual screening. Chem. Sci. 13 (4), 1060–1079. 10.1039/d1sc05613a 35211272 PMC8790893

[B41] Xu X. Wang W. (2022). Orthogonal experiment to optimize the extraction process of sophoraside from Sophora japonica. Asia Pac Tradit. Med. 18 (5), 75–77. 10.20062/j.cnki.APTM.2022.05.001

[B42] Zhou S. Ai Z. Li W. You P. Wu C. Li L. (2020). Deciphering the pharmacological mechanisms of taohe-chengqi decoction extract against renal fibrosis through integrating network pharmacology and experimental validation *in vitro* and *in vivo* . Front. Pharmacol. 11, 425. 10.3389/fphar.2020.00425 32372953 PMC7176980

