# Supplementary material for: Investigation of Fructus sophorae extract’s therapeutic mechanism in atrophic vaginitis based on network pharmacology and experimental validation
Source: Front Pharmacol. 2025 May 8;16:1571976. doi: 10.3389/fphar.2025.1571976 (PMC12095276; doi:10.3389/fphar.2025.1571976)
Supplement: Supplementary file 1 [file DataSheet1.zip › original data/Network pharmacology section raw data.docx]

3.1 Results of a Network Pharmacology Study of FS for the Treatment of AV

3.1.1 Identification of FS drug targets

Tab.1 Screening of active ingredients of FS

| [Molecule Name](https://old.tcmsp-e.com/tcmspsearch.php?qr=Fructussophorae&qsr=herb_en_name&token=cddb1e5ad8b51acab644da84357f4986) | [MW](https://old.tcmsp-e.com/tcmspsearch.php?qr=Fructussophorae&qsr=herb_en_name&token=cddb1e5ad8b51acab644da84357f4986) | [AlogP](https://old.tcmsp-e.com/tcmspsearch.php?qr=Fructussophorae&qsr=herb_en_name&token=cddb1e5ad8b51acab644da84357f4986) | [Hdon](https://old.tcmsp-e.com/tcmspsearch.php?qr=Fructussophorae&qsr=herb_en_name&token=cddb1e5ad8b51acab644da84357f4986) | [Hacc](https://old.tcmsp-e.com/tcmspsearch.php?qr=Fructussophorae&qsr=herb_en_name&token=cddb1e5ad8b51acab644da84357f4986) | [OB (%)](https://old.tcmsp-e.com/tcmspsearch.php?qr=Fructussophorae&qsr=herb_en_name&token=cddb1e5ad8b51acab644da84357f4986) |
| --- | --- | --- | --- | --- | --- |
| [baicalein](https://old.tcmsp-e.com/molecule.php?qn=2714) | 270.25 | 2.33 | 3 | 5 | 33.52 |
| [Vetol](https://old.tcmsp-e.com/molecule.php?qn=3518) | 126.12 | -0.57 | 1 | 3 | 53.23 |
| [sophocarpine](https://old.tcmsp-e.com/molecule.php?qn=3627) | 246.39 | 1.39 | 0 | 3 | 64.26 |
| [Sophoricoside](https://old.tcmsp-e.com/molecule.php?qn=3637) | 432.41 | 0.16 | 6 | 10 | 10.42 |
| [kaempferol](https://old.tcmsp-e.com/molecule.php?qn=422) | 286.25 | 1.77 | 4 | 6 | 41.88 |
| [genistin](https://old.tcmsp-e.com/molecule.php?qn=480) | 432.41 | 0.16 | 6 | 10 | 13.35 |
| [genistein](https://old.tcmsp-e.com/molecule.php?qn=481) | 270.25 | 2.07 | 3 | 5 | 17.93 |
| [3,4,5-trihydroxybenzoic acid](https://old.tcmsp-e.com/molecule.php?qn=513) | 170.13 | 0.63 | 4 | 5 | 31.69 |
| [matrine](https://old.tcmsp-e.com/molecule.php?qn=5944) | 248.41 | 1.42 | 0 | 3 | 63.77 |
| [Cytisine](https://old.tcmsp-e.com/molecule.php?qn=5945) | 190.27 | -0.34 | 1 | 3 | 69.4 |
| [stizolamine](https://old.tcmsp-e.com/molecule.php?qn=5947) | 197.23 | -2.17 | 5 | 7 | 67.52 |
| [quercetin](https://old.tcmsp-e.com/molecule.php?qn=98) | 302.25 | 1.5 | 5 | 7 | 46.43 |

Tab.2 Target screening of the active ingredients of FS

| Common name | Common name | Common name | Common name | Common name | Common name |
| --- | --- | --- | --- | --- | --- |
| TYR | ALOX12 | AMY1A | DPP7 | PLA2G2A | AXL |
| DAO | PTPRS | GRK6 | SIGMAR1 | PLA2G4A | NUAK1 |
| ERN1 | ADORA2A | TERT | DPP4 | CYP2C9 | AKR1C2 |
| CA2 | CDK5R1 CDK5 | MAPT | CHRNA4 | KCNH2 | AKR1C1 |
| CA7 | CCNB3 CDK1 CCNB1 CCNB2 | TBXAS1 | SLC6A3 | CYP2C19 | AKR1C3 |
| CA1 | ARG1 | MGAM | HTR3A | HTR1A | AKR1C4 |
| CA3 | DAPK1 | HTR2A | HTR1B | ADRA2B | AKR1A1 |
| CA6 | MPG | HTR2C | HTR1D | CYP2D6 | APP |
| CA12 | SLC22A12 | ESRRB | DPP8 | PRMT3 | PARP1 |
| CA14 | AKR1B10 | ALDH2 | FAAH | NOS2 | MMP12 |
| CA9 | TNKS2 | MIF | DPP9 | NAE1 | CD38 |
| FUT7 | TNKS | CBR1 | HDAC3 | KIT | TOP1 |
| CA4 | CDK6 | IL2 | HDAC6 | OPRD1 | ESR1 |
| CA5B | CDK2 | PTGS1 | HDAC2 | CYP1A1 | PTGS2 |
| CA5A | CYP19A1 | SLC6A2 | NCOR2 HDAC3 | PIK3CG | CFTR |
| CA13 | CSNK2A1 | MAOB | METAP1 | TOP2A | PFKFB3 |
| SQLE | EGFR | PON1 | HDAC8 | INSR | ABCC1 |
| LDHA | AVPR2 | STS | HDAC11 | MYLK | HSD17B1 |
| LDHB | F2 | IGFBP3 | HDAC10 | APEX1 | AHR |
| TTR | PIM1 | MCL1 | CHRM4 | ST6GAL1 | ESRRA |
| IGF1R | AURKB | SNCA | CHRM5 | FYN | ABCB1 |
| ALK | DRD4 | TLR9 | CHRM3 | TACR2 | CYP1B1 |
| SERPINE1 | MPO | CRHR1 | DRD1 | PRKDC | ABCG2 |
| ESR2 | PIK3R1 | PPARA | MMP8 | MAPK3 | ADORA1 |
| BCL2L1 | PYGL | PTPN1 | ADH1A | SIRT1 | ACHE |
| GPR35 | SRC | PLAT | ADH1C | SRD5A2 | MAOA |
| COMT | PTK2 | F10 | MME | HSD17B7 | GLO1 |
| TPMT | KDR | PLAU | CHRNB1CHRN1  CHRNG CHRND | NAAA | SYK |
| NUDT1 | MMP13 | IGFBP6 | CHRNA3 | HSD11B1 | GSK3B |
| YARS | MMP3 | IGFBP4 | HTR2B | PAOX | MMP9 |
| FUCA1 | PLK1 | IGFBP5 | DRD2 | GRM5 | MMP2 |
| ADORA3 | CDK1 | IGFBP2 | DRD3 | SRD5A1 | ALOX15 |
| MAP2K7 | PKN1 | IGFBP1 | PNP | ITGA2B ITGB3 | HSD17B2 |
| ANPEP | MET | BAD | KISS1R | CTSK | BACE1 |
| GBA | NEK2 | TNF | PNMT | CTSL | EPHX1 |
| NOX4 | CXCR1 | CHRNA4 CHRNB2 | KDM4E | CTSB | CALM1 |
| AKR1B1 | CAMK2B | CHRNA3 CHRNB4 | IKBKB | RNPEP | EPHX2 |
| XDH | AKT1 | CHRNA7 | NTRK2 | DNPEP |  |
| FLT3 | NEK6 | DNM1 | AR | CHRM2 |  |
| ALOX5 | PLA2G1B | BCHE | LCK | CHRM1 |  |

3.1.2 Identification of disease targets

Tab.3 Information about potential target proteins for AV

| Gene Symbol | Gene Symbol | Gene Symbol | Gene Symbol | Gene Symbol | Gene Symbol | Gene Symbol |
| --- | --- | --- | --- | --- | --- | --- |
| ERCC8 | MIAT | CTSD | TXN | PVT1 | HTR2A | LPO |
| CSH1 | MIR26B | WRN | ACTC1 | XIST | MIR148A | DNAJC21 |
| CSH2 | MIRLET7I | CD274 | IFNG | CST3 | GAPDH | CTNNA1 |
| CYP19A1 | LINC01772 | NTRK1 | MBL2 | SOD2 | CASP3 | CEP63 |
| HSPA9 | PIK3CA | HLA-DRB1 | MIR193A | CHGA | CALCR | ARRB2 |
| TNF | MIR93 | CTNNB1 | COL3A1 | PON1 | MIR377 | WWTR1 |
| APOE | CSF1R | LINC01672 | IL1R1 | TGFB1 | MIR331 | SCGB2A1 |
| IL6 | INSR | CTSB | S100B | UCHL1 | TKT | NODAL |
| OXT | FGFR2 | CCL3 | MIR195 | MAP2 | TNFRSF1A | IGF2BP3 |
| CXCL8 | SHBG | IL1RN | MIR29C | MIRLET7G | RPS6KB1 | FGFR4 |
| ESR1 | MIR320A | CRH | PTPN1 | MMP2 | HSPA8 | TNFRSF25 |
| TTR | KIT | ACHE | MIR140 | FBN1 | GSK3B | ZIC2 |
| IL1B | IL17A | TMX2-CTNND1 | MIR335 | PHF1 | TLR7 | LOXL4 |
| TP53 | EGFR | AKT1 | MIR30A | MAPK1 | APEX1 | TIAM1 |
| ESR2 | SOD1 | ICAM1 | PTPRC | A2M | PCNA | ELAVL2 |
| IL1A | MIR363 | GAS5 | MMP8 | ACE | HIF1A | VPS54 |
| IL10 | PSEN2 | MKI67 | CYP17A1 | OGN | MEG3 | NF2 |
| TLR4 | SYP | BDNF | LTF | GAL | IGFBP1 | CCL28 |
| GRN | MIR29B1 | GSTT1 | CYP21A2 | POMC | XPC | CX3CL1 |
| FN1 | HMCN1 | CLU | TIMP1 | MIR206 | VCAM1 | HS6ST1 |
| NOS3 | H19 | PRL | FBLN5 | MIR657 | CBS | S100A12 |
| EPHA2 | GSTM1 | GFAP | IGF1 | LOC126862549 | CYP2D6 | TTC21B |
| MIR29A | MAPK8IP1 | VEGFA | MIR143 | BRAF | IGF1R | ADCY10 |
| TLR2 | GNRH1 | MIR223 | ELN | COMT | DRD2 | CHRNB4 |
| BACE1-AS | IL4 | CP | HNF1A | MIR100 | CXCL1 | MLN |
| AR | MIR125A | NPY | VIP | RHOA | IVL | MYBPC1 |
| MIR210 | ALB | MMP1 | KRAS | BDNF-AS | RETN | IFIT1 |
| CD4 | ABCB1 | MIR21 | MIR127 | MIR155 | MIR320B1 | CAMTA2 |
| MIR146A | UNC5C | MIRLET7D | MIR200B | IL13 | ERCC6 | SIGLEC1 |
| CDKN2A | BRCA1 | PIK3R1 | MIR485 | NOS2 | ENO2 | FKBP4 |
| CCR6 | VIM | IL2 | MIR361 | CALB2 | MMP7 | UTRN |
| GBA1 | CHAT | CXCL12 | COL1A1 | AGER | OGG1 | LGALS1 |
| SLPI | PRNP | PPARG | MTOR | CALCA | IL7 | ZDHHC24 |
| MIR106B | BCL2 | G6PD | ECE1 | SRC | STAR | B3GALT4 |
| BCYRN1 | BBS10 | CCL2 | MLANA | CCNE1 | EGF | NEB |
| PTEN | INS | LINC02605 | LIPC | CD80 | AKT2 | B4GALT6 |
| CCL5 | MME | TGFB2 | SST | MAPK3 | IAPP | PLXNC1 |
| MPO | CD8A | LIF | GSTP1 | NLRP3 | CXCR4 | GZMM |
| CDKN2B-AS1 | CSF2 | PEPD | TH | IGFBP3 | KDR | KLHL41 |
| HRAS | MAP2K1 | CERNA3 | IGF2 | BGLAP | TERC | OTUD7A |
| SERPINA3 | TP63 | PRKCA | MSR1 | TLR3 | AOC3 | RMI2 |
| PLAU | HSPA4 | MIR142 | TSPO | CCK | MIR27A | SNRNP27 |
| MIR34A | SLC25A24 | CRP | ERCC2 | GALK1 | HLA-B | FAAP20 |
| TF | PTGS2 | TMEM106B | TSC2 | ITGB2 | MAPK14 | MIR27B |
| CCR5 | MIR126 | ITIH4 | DICER1 | GAD1 | SELE | ALMS1 |
| MIR22 | MMP9 | H2AC18 | NGF | SERPINE1 | SREBF1 | HOXA2 |
| MIR15A | HNF1B | CAPN2 | SIRT1 | PVALB | MIR125B1 | SSPN |
| PGR | ABCA7 | HLA-DQB1 | HPD | MIR9-1 | MIRLET7A1 | VTCN1 |
| CCL4 | MIR424 | PRKN | TMEM121B | MIR34C | MIR199A1 | IL16 |
| SOX2-OT | AMH | MIR30E | FSBP | CD40 | PARK7 | SLC36A1 |
| HSPB8 | PECAM1 | HSD11B1 | SOCS3 | TROAP-AS1 | HNF4A | MTMR2 |
| CFTR | MIR196A1 | HSD11B2 | PTGER4 | MIR200C | MIR145 | F2R |
| SELL | DPP4 | TNFRSF10A | RNASE3 | DSEL | MIR214 | MIR135B |
| ANXA2 | SERPINA6 | PDCD1 | TAC1 | FBXO32 | EDN1 | UPK3A |
| CR1 | SLC6A4 | RAD51 | UBXN11 | MYC | SERPINC1 | ADAR |
| MAP3K5 | SLC5A2 | FLG | MIR4516 | SALL1 | CAPN1 | KLRK1 |
| FGF1 | SLC5A4 | MIR23B | PWAR1 | CFAP47 | CSF1 | IL2RG |
| DIABLO | MDM2 | MIR130B | YY1 | MIR149 | C1S | PHLDA2 |
| CCR1 | S100G | RAB3GAP2 | LHFPL5 | FERMT1 | NPPB | HELLS |
| FGF2 | ITGA2 | MMP12 | CSNK1A1 | KLK3 | LEP | TP53BP1 |
| TERT | TMC8 | IFNGR1 | SLC40A1 | FLCN | GRB2 | CHKA |
| THBS1 | SOD2-OT1 | GHSR | UBE2K | MRE11 | CHRM1 | CCBE1 |
| PLD2 | SMAD4 | PIK3CB | APPBP2 | MYH11 | RECQL4 | LINC01194 |
| MET | SLC18A3 | DNAH8 | EDN3 | CTSA | MIR15B | PRKCH |
| BMPR1A | TFAP2A | IL3 | CPT2 | MIR199B | XPA | CMA1 |
| HMGA1 | CD55 | ZNF664 | GPT | ANOS1 | RARB | PIGN |
| UBQLN1 | TIMP3 | IL17RC | SERPINA12 | MIR191 | MIR185 | GRID2 |
| HTR2C | CSN1S1 | ALAD | CLSTN3 | TCOF1 | PIEZO1 | WARS2 |
| CAST | VEGFC | PDE6A | PHGDH | MUTYH | CDH1 | PCLO |
| F8 | CPQ | C4A | ASS1 | PROM1 | NGFR | EBF3 |
| CREBBP | SOX9 | PDGFC | PGM1 | CD151 | GCG | FDXR |
| CHRNA4 | VEGFD | PRPF31 | SIGMAR1 | BRIP1 | STAT1 | NARS2 |
| P4HB | FST | PLEKHA1 | SUCLA2 | BIRC5 | CDKN3 | POGZ |
| DRD1 | GPX3 | RAD51B | ARID1B | CD34 | OPRM1 | DVL1 |
| IDO1 | CDH5 | BCO2 | GAS1 | CYB5A | NEFL | CEP83 |
| CD59 | MIR184 | USH2A | AGFG1 | AMACR | CD79A | AURKA |
| CEP290 | B3GLCT | EYS | CTSE | ITGAL | ATM | MYO18B |
| PDE4A | CSF3 | RP1L1 | EDIL3 | NKX2-1 | MIR182 | CD38 |
| CASP9 | BMP4 | MX1 | MARCO | FUT2 | LCN2 | SLX4 |
| MIR425 | TNFSF13B | CDK4 | NPAS3 | BLM | MIR150 | SEPTIN7 |
| HSPG2 | IL2RA | CASP7 | TRPV2 | IFNB1 | MIF | POLR3K |
| CD68 | CTLA4 | CCNB1 | STARD13 | CTBP2 | SMAD3 | COL9A1 |
| PLCG1 | HSPB1 | BECN1 | SYPL1 | XK | APOB | CDC42 |
| SOCS1 | TRH | ITGA6 | VPS13D | NDE1 | MIR17 | CCKBR |
| LAMA3 | CASR | TNFSF10 | CAVIN4 | FANCA | MIR181A1 | MUC5AC |
| AXIN2 | DNTT | EWSR1 | FAM240A | FANCG | MIR486-1 | ALDH1A3 |
| LINC-ROR | SP1 | FILIP1L | CASC18 | FANCE | FGFR1 | FOXC1 |
| COL18A1 | PTHLH | GOT1 | LIFR | FANCF | IRS2 | CYP26A1 |
| WNT5A | NAT2 | KRT10 | PPARD | MUC5B | LOX | MECOM |
| PEBP1 | BCL2L1 | CPE | ADM | PAEP | ERCC4 | MIR124-2 |
| RAC1 | SLCO1B1 | SELP | SOD3 | CSGALNACT2 | COL1A2 | FAAP100 |
| ICOSLG | EFEMP1 | WNT3A | SCARNA5 | PRKD1 | MIR19B1 | PROK2 |
| FMR1 | TGIF1 | SOST | HDAC6 | WWOX | CTNND2 | PROKR2 |
| LPL | ESRRA | LGR4 | TUBB | TSC1 | MTHFR | AHI1 |
| PRDX2 | CXCL2 | XYLT2 | PXN | CHRM3 | ERCC1 | CSPP1 |
| HGF | GHRL | FABP2 | NFIB | MT-CYB | LMNA | B9D1 |
| AMBP | CYP27B1 | CSK | SERPINB2 | MIRLET7C | STH | KIAA0586 |
| SLC2A3 | SDHB | ACP5 | S100A6 | MAPKBP1 | GALR1 | KATNIP |
| IGFBP2 | CCN2 | UGT2B17 | ENO1 | HOXA10 | MIR23A | MC2R |
| FAS | CCR2 | IBSP | DSP | BSG | EMSLR | HCN4 |
| APOA1 | TWIST2 | NPHS1 | ANGPTL4 | ZEB2 | NOTCH1 | SLC1A1 |
| ERCC5 | HDAC9 | SCD | FABP4 | CCND1 | SAA1 | ERN1 |
| CD86 | LORICRIN | EIF2AK3 | UCP1 | MIR375 | CTRL | PPP1CB |
| CRHR1 | PIK3CG | NME1 | IL23A | ESRRB | XRCC1 | ITPR3 |
| VDAC1 | ADIPOQ | UBE3B | TLX1NB | KRT16 | CHRM2 | MDH1 |
| FBN3 | SMAD2 | CD28 | TFPI | KIF1B | MIR211 | GALC |
| SLC1A3 | MIR139 | F2 | AZU1 | GZMB | CYP3A4 | CNTFR |
| SYT1 | KRT18 | STK32B | TDO2 | KIF7 | ABCA2 | GART |
| GRK2 | CYP2C9 | EPHA1 | PRSS1 | EDNRA | IRS1 | TICAM1 |
| MTR | CYP2C19 | XAB2 | ITGA1 | CBL | NPC1 | DTNBP1 |
| OGDH | CASP8 | MS4A4A | MIR208B | PTCH1 | SNCG | PABPC1 |
| CHRNB2 | IGFBP5 | MS4A6E | TRA-TGC7-1 | NOD2 | MIR146B | DSCAM |
| HRH1 | LTA | DDR1 | P2RX4 | MIR376A1 | MIR221 | TNFRSF6B |
| GSTO1 | ICAM2 | FOXO3 | GAST | GAB1 | ITGB4 | DPYSL3 |
| NEFM | MIR20A | PTN | COL7A1 | BMP7 | CYCS | RNF19A |
| RGS4 | CD40LG | PLK4 | TNXB | CAPN14 | CDK1 | ADARB2 |
| SERPINE2 | RMRP | CENPE | KRT7 | FCGR1A | TNFRSF1B | HLA-DMA |
| F3 | SYK | CEP152 | MKKS | FGF7 | MIR130A | HLA-DMB |
| GDNF | MB | COG2 | S100A8 | NLRP1 | ABCA1 | TOMM20 |
| MYO18A | APCS | RECQL | HSPD1 | IFI27 | KLK6 | HAS1 |
| DDB1 | ENPEP | CD36 | AIRE | CARS2 | EPO | SOHLH1 |
| LEPQTL1 | MED12 | MIR138-1 | MUC1 | GNRHR | CAV1 | TMBIM4 |
| MMP3 | SRF | CDR1-AS | TGFBR2 | DPYD | XIAP | EPHB4 |
| ERBB4 | IGFBP4 | LPAR1 | HOTAIR | EVC2 | TLR9 | CTTN |
| ABL1 | IL11 | AGPAT2 | WT1 | WNT4 | MIR342 | CXADR |
| EIF4E | CD44 | BSCL2 | ERBB2 | FHIT | MIR144 | PRPF3 |
| NRG1 | GPC1 | PRTN3 | TGFA | NCAM1 | MIR532 | KIR2DS4 |
| ATP7A | VDR | CAMK1D | MALAT1 | TOP2A | NOTCH3 | HERC2 |
| CASP2 | LAMC1 | CD83 | FBXW7 | CDKN1A | MIR497 | SLC6A5 |
| STMN1 | MIRLET7B | C3 | DES | CYP3A5 | MIR212 | MAMLD1 |
| TPH1 | AHR | RXRA | U2AF1 | PLK1 | NFKBIA | GDPD2 |
| MIR98 | PZP | COL4A4 | FIG4 | NF1 | CASP1 | SLC6A9 |
| F13A1 | MIR193B | HAMP | TLR1 | MUC6 | JUN | TBXAS1 |
| LAMC2 | MIR423 | MEF2A | PPP2R1A | SH2D1A | CDKAL1 | HADH |
| LAMB3 | JAZF1 | IL9 | MIR200A | CCR7 | CAT | VRK1 |
| CDKN2B | LEPR | TGM1 | KRT20 | GATA4 | IL18 | SAMHD1 |
| COL17A1 | MIR186 | F7 | ADA | IL18R1 | HCRT | PDGFRL |
| EXT2 | ELANE | NFATC1 | RB1 | MKS1 | CXCL10 | EPHA6 |
| GJB2 | ODC1 | TNFRSF11A | COL12A1 | AMOTL1 | NQO1 | RNF125 |
| ADAM17 | SEMG1 | CYP24A1 | CYP11A1 | TLR5 | ERCC3 | ANKH |
| PLAT | ATXN1 | RAP1A | BRCA2 | HBG2 | PLG | RSPH4A |
| FASLG | EPM2A | SLC26A2 | RET | CDK2 | IGHE | ASB10 |
| LOXL1 | RETREG1 | CA10 | CCL20 | MIR22HG | MIR204 | CCDC90B |
| COL25A1 | CA2 | WNT16 | MIR222 | FAM215A | MIR338 | ZNF608 |
| KL | TRE-TTC3-1 | THSD7A | MIR129-1 | MIR376C | PPARA | GRAMD2B |
| ZFAS1 | KITLG | AGT | MIR31 | IFI16 | TBP | ZNF81 |
| NFKB1 | VPS13C | PLA2G7 | GATA3 | PLB1 | GAP43 | C16orf82 |
| GUSB | SCT | PBX1 | NEAT1 | ARG1 | HBA1 | IL2RB |
| F5 | CD46 | NR3C2 | NLRP7 | CYP11B1 | ADCYAP1 | BLK |
| NR3C1 | FGF9 | NCOA1 | BBS12 | MAF | GALT | ITGAV |
| LGALS4 | SLC2A1 | PYY | MIR141 | ITGA3 | MIR134 | REL |
| PLAGL1 | MT-ATP6 | EDNRB | MIR92A1 | PI4KA | CCL11 | THY1 |
| PAX8 | BGN | ATF2 | MYOG | IL17RA | PCSK1N | CXCR5 |
| POU5F1 | FGF23 | PRKAR1A | BBS1 | ESRRG | CALR | NBR1 |
| FOXP1 | SPP1 | COL2A1 | FOXP3 | PAPPA-AS1 | MIR874 | PABPN1 |
| MOXD1 | STAT3 | SMAD9 | FREM1 | RNU12 | B2M | UGT1A6 |
| MSI2 | GAA | GDF11 | MSH2 | LGALS3 | GJA1 | BOK |
| GCNT1 | ADAMTS2 | SOX6 | LRRC56 | RASSF1 | CCNA2 | TPP2 |
| MKLN1 | AGTR1 | MYD88 | CGA | KRT12 | APOH | PHB1 |
| SLCO4C1 | TLR8 | JAK1 | CEACAM5 | SRY | MIR30B | DCK |
| XPO4 | POU4F1 | PARP1 | EZR | PGR-AS1 | MIR574 | OLIG2 |
| CARD19 | REN | LGMN | SPINK5 | CD69 | MIR151A | CEACAM1 |
| SERPINH1 | DNMT1 | F9 | PGC | DNMT3A | MIR455 | SPINT1 |
| RAG2 | MMP13 | SPINK1 | UCA1 | MCL1 | FSHR | CDH3 |
| ANXA1 | HSP90AA1 | JAK2 | TFF1 | NPHP1 | PRMT7 | IL4R |
| CD1A | ITLN1 | PTK2B | CAPN15 | SPTAN1 | CXCR3 | WNK1 |
| DDC | CHI3L1 | SPARC | SNRPE | CYP4V2 | TG | CXCL5 |
| CD163 | MIR25 | SMAD7 | MIRLET7E | TTC27 | CREB1 | GRIK2 |
| TRAPPC10 | SHC1 | ENO3 | HLA-C | F11 | TCF7L2 | HEXB |
| CD1C | RNF217-AS1 | ANK3 | SMAD5-AS1 | POLD1 | BAX | ATP2B3 |
| ZNRF3 | ROM1 | C4BPA | MIR494 | CD209 | PTPN22 | LETM1 |
| KLK7 | HMGB1 | KCNQ4 | BARD1 | ACTN2 | SLC2A4 | LMX1B |
| ATRX | IL6R | SERPINI2 | PAX2 | EPCAM | IL1RAPL2 | SLC44A1 |
| DMBT1 | CISH | SNAP23 | ERBB3 | DOCK8 | PTGS1 | AGTPBP1 |
| MMUT | TNFRSF11B | MSMB | APC | MTMR10 | ACTB | EXOSC9 |
| SKP2 | CACNG5 | GNAS | NEU1 | IL36G | HTR1A | FGF14 |
| IL24 | FBN2 | IL33 | UBE3A | CYP1B1 | NFE2L2 | PIGT |
| DPH1 | FGFR3 | KRT5 | CIITA | RAG1 | WNT3 | MFF |
| JUP | VWF | PSMB8 | MIR374A | PORCN | GHR | PITRM1 |
| PSMC4 | MIR483 | KCNJ6 | NPHP4 | IL15 | GH1 | RUBCN |
| MRTFA | MIR133A1 | RPA1 | IFNA2 | CKAP2 | MAPK10 | GEMIN5 |
| MIR101-1 | ANXA5 | SLC6A1 | LUCAT1 | BRD2 | CYP1A1 | MRM2 |
| MIR106A | KCNQ1 | ATP6V0A2 | MIR498 | MIR1251 | BLVRB | SLC25A46 |
| GDF15 | FTMT | CHD2 | MIR637 | LYVE1 | IL5 | LYRM7 |
| CHRNE | ABCC6 | SLC2A10 | MIR572 | RHOT1 | GC | SH3TC2 |
| CDC45 | CD63 | RTEL1 | SPRY4-IT1 | TBX3 | TGM2 | ARMC9 |
| BTD | ANGPT2 | STN1 | LOC126806658 | DHCR7 | MIR148B | RTTN |
| IL12A | TNFSF11 | HNMT | CDKN1B | S100A7 | MIR330 | CDC20 |
| SLC35A2 | MYOD1 | KANSL1 | MSH6 | PMM2 | MIR124-1 | FGF4 |
| KCNMA1 | MIR451A | TCEA3 | PMVK | ENG | MIR24-1 | BNIP3 |
| PRLR | SCARB1 | LINGO2 | KRT19 | GRP | RHOD | HSD3BP4 |
| TCTN2 | OPTN | WHRN | STAT6 | FGF8 | CYP2E1 | ZFYVE26 |
| MMP26 | CHCHD10 | SOX10 | MLH1 | DUS3L | BMP2 | ATP2A2 |
| NKX6-2 | ARMS2 | DSTYK | RELA | VMAC | FLT1 | SLC31A1 |
| CYBB | BAD | CETN2 | GLI3 | CEACAM3 | SERPINA7 | UBA5 |
| EPG5 | PCSK9 | RAF1 | TIMP2 | TRPV4 | IFNA1 | UFM1 |
| IL22 | SERPINA1 | ATR | AREG | KRT83 | TMPO | S100A9 |
| EYA1 | ACACA | PDGFRB | NR5A1 | KRT1 | APLP2 | PAK1 |
| HPSE | DNAJC6 | NPM1 | ITGAM | BIRC3 | MAPK8 | CRHR2 |
| PNPLA8 | COQ2 | TYMS | STK11 | TP73 | MIR18A | LTBP2 |
| ACAN | CSTA | SNAI2 | PALB2 | MFN2 | RBFOX3 | RXRB |
| RXYLT1 | TRP-AGG2-5 | SMARCA4 | VTRNA1-1 | PAX7 | PGF | IL1RL1 |
| BCL6 | NOG | RAD52 | TRIM32 | DNMT3B | AATF | SMTN |
| JAM3 | TEK | LIPE | HLA-DQA1 | EHMT2 | HP | HMBS |
| ADPRH | SLC5A1 | ADRB1 | SHH | AGXT2 | LRP2 | FANCL |
| ITGB1 | MIR10A | LPIN1 | PMS2 | MTM1 | LACTB | SELENBP1 |
| PRICKLE2-AS1 | MIR19A | TPO | WNT10A | MICA | DRD4 | RBP1 |
| SIX5 | PTX3 | PYGM | MIR10B | TAC3 | GPD2 | SLC26A9 |
| SLC6A20 | ALPP | NRF1 | BBS7 | LHCGR | P4HA2 | FCGR2A |
| CEBPB | AXIN1 | GRB14 | MIR551A | MSX1 | SLC30A8 | HEXA |
| IRF3 | FASN | DSG1 | TSHR | PAOX | CYP1A2 | ACADVL |
| MMP10 | RPGR | ST3GAL4 | FANCM | MGMT | CTSL | CXCL9 |
| ACP3 | RCVRN | SRSF6 | BBS5 | HDAC1 | FPR2 | CHGB |
| TFEB | XDH | CCL17 | CHRNG | E2F1 | CHRNA7 | NOBOX |
| CA9 | PAX6 | EVPL | TNFRSF10B | ZMIZ1 | OLR1 | SPRR1A |
| SOCS2 | RPE65 | PPARGC1B | PFN1 | MATN1 | RCAN1 | GSTA1 |
| ACVR1 | MERTK | MTUS1 | FRAS1 | CBLIF | CXCR2 | KTN1 |
| GPT2 | CFI | MTUS2 | CHEK2 | RPGRIP1L | KRT14 | NKX3-1 |
| FOXD1 | NPPA | SH3PXD2A | MIR381 | HMX1 | TJP1 | SLC2A12 |
| FANCI | PAX3 | CCR8 | MIR205 | SAT1 | SGK1 | RASSF10 |
| SETD2 | TPPP3 | RHEB | MIR429 | ORM1 | EP300 | MIR100HG |
| DROSHA | GABRR1 | SERPINF2 | INHA | HSD17B4 | HSPA1A | LINC01162 |
| ELAVL1 | ITGB3 | AGTR2 | SRD5A1 | GATA6 | LTBP1 | SIRT2 |
| TMEM216 | CX3CR1 | PLEK | DYNC2I2 | MUC2 | CTNNA3 | GFRA1 |
| DLEU1 | TGFBR1 | CTSK | DYNC2I1 | ATG5 | GCK | NEDD4 |
| DPP6 | SYNGAP1 | SRPX | POLR1C | MYH3 | ABCC8 | ETV5 |
| PPIF | ADNP | THBD | MIR99A | RAB11A | KCNJ11 | XYLT1 |
| TBC1D20 | NTHL1 | DUSP1 | PKP1 | ATIC | PDX1 | STING1 |
| ITGA4 | FCGR3A | ISL1 | FANCD2 | ARX | WFS1 | STAT5A |
| CCL7 | RAB3GAP1 | PCBD1 | RAD51C | TBX5 | NEUROD1 | FSCN1 |
| CTSF | RBBP8 | GAD2 | RAD51D | PNPLA6 | PAX4 | SERPINB5 |
| ADSL | NR1H2 | UMOD | KRT8 | GHRH | HCCAT5 | CLDN4 |
| DPM1 | PTK2 | NES | SCAF4 | PHOX2B | PDE5A | CLDN3 |
| SUMF1 | HBEGF | AHSP | FANCC | RBM28 | HSPB2 | CTAG1B |
| SYNE1 | PTH | ACSS3 | SMO | HRH2 | CDH2 | RNF216 |
| SIGLEC5 | FOS | BAK1 | MYH10 | HIPK2 | CRYBB1 | HPGD |
| TRPM3 | CLSTN2 | CRAT | KRT4 | KHSRP | TM2D1 | TSLP |
| PLCL1 | PAX5 | STC2 | PIK3C2A | HLA-DRB5 | VTN | HOTTIP |
| PPP1R14C | MSH3 | SLC12A3 | TGFB3 | KCNT2 | SLC17A5 | ARF1 |
| TMEM63B | NAMPT | TRPV1 | FOXL2 | HLA-DQA2 | ADRB2 | SFTPD |
| GPSM3 | RUNX2 | APAF1 | FREM2 | ZNF557 | MAP3K3 | OSBPL8 |
| HLA-DRB6 | TNNT2 | NECTIN2 | JAK3 | SDHD | UCP2 | ALPG |
| ENSG00000226334 | HBA2 | PLAUR | NECTIN1 | TACR3 | PON2 | TNC |
| OSM | DNASE1 | PLIN3 | TMEM67 | CHD7 | DCN | CEL |
| ADAMTS20 | PLIN2 | MBP | LAMA5 | EIF4EBP1 | CCR3 | NUCB2 |
| BTNL2 | MMP14 | BCL2L11 | KRT25 | SNRNP70 | CDX2 | SNORD24 |
| LGR6 | BMP6 | APH1B | TYMP | DMD | FURIN | SNORD44 |
| MYH6 | CLUAP1 | IKBKB | EPHA4 | POLR1D | NOS1 | INHBA |
| CARMN | PNPLA3 | GGT1 | CFAP410 | TCTN3 | NTRK2 | AP1G1 |
| KRT17 | FOXO1 | TFRC | SNHG29 | RARA | CCDC88A | SDC1 |
| NRTN | SORBS2 | CEP164 | TENM3 | PRICKLE2 | WDR90 | LINC01554 |
| DAPK1 | LILRA2 | RECQL5 | CPLANE1 | NHLRC2 | HCG25 | MIR16-1 |
| LGR5 | PECR | SHISA6 | TPRN | VAC14 | LAMB2 | TRV-AAC1-4 |
| ASTN2 | CDH19 | NOTCH4 | CWC15 | PCDH10 | RNASEH2A |  |

3.1.3 Identification of therapeutic targets for FS against AV and PPI network construction

Tab.4 Targets with Degree ≥ 20 in the PPI network of key targets for AV treatment with FS

| Name | Degree | Name | Degree | Name | Degree |
| --- | --- | --- | --- | --- | --- |
| TNF | 65 | BCL2L1 | 35 | PPARA | 26 |
| AKT1 | 65 | GSK3B | 34 | PIK3R1 | 25 |
| ESR1 | 60 | SERPINE1 | 34 | ESR2 | 25 |
| EGFR | 54 | MCL1 | 33 | MMP3 | 25 |
| MMP9 | 53 | KDR | 32 | CTSB | 25 |
| PTGS2 | 47 | IGF1R | 31 | PLAU | 24 |
| SRC | 47 | IL2 | 29 | CYP19A1 | 23 |
| MAPK3 | 41 | ABCB1 | 28 | CDK2 | 23 |
| SIRT1 | 38 | AR | 27 | PTPN1 | 21 |
| MMP2 | 38 | KIT | 26 | CDK1 | 20 |
| PARP1 | 35 | MET | 26 | IKBKB | 20 |
