# Supplementary figures and images for: Investigation of Fructus sophorae extract’s therapeutic mechanism in atrophic vaginitis based on network pharmacology and experimental validation
Source: Front Pharmacol. 2025 May 8;16:1571976. doi: 10.3389/fphar.2025.1571976 (PMC12095276; doi:10.3389/fphar.2025.1571976)

Western blot


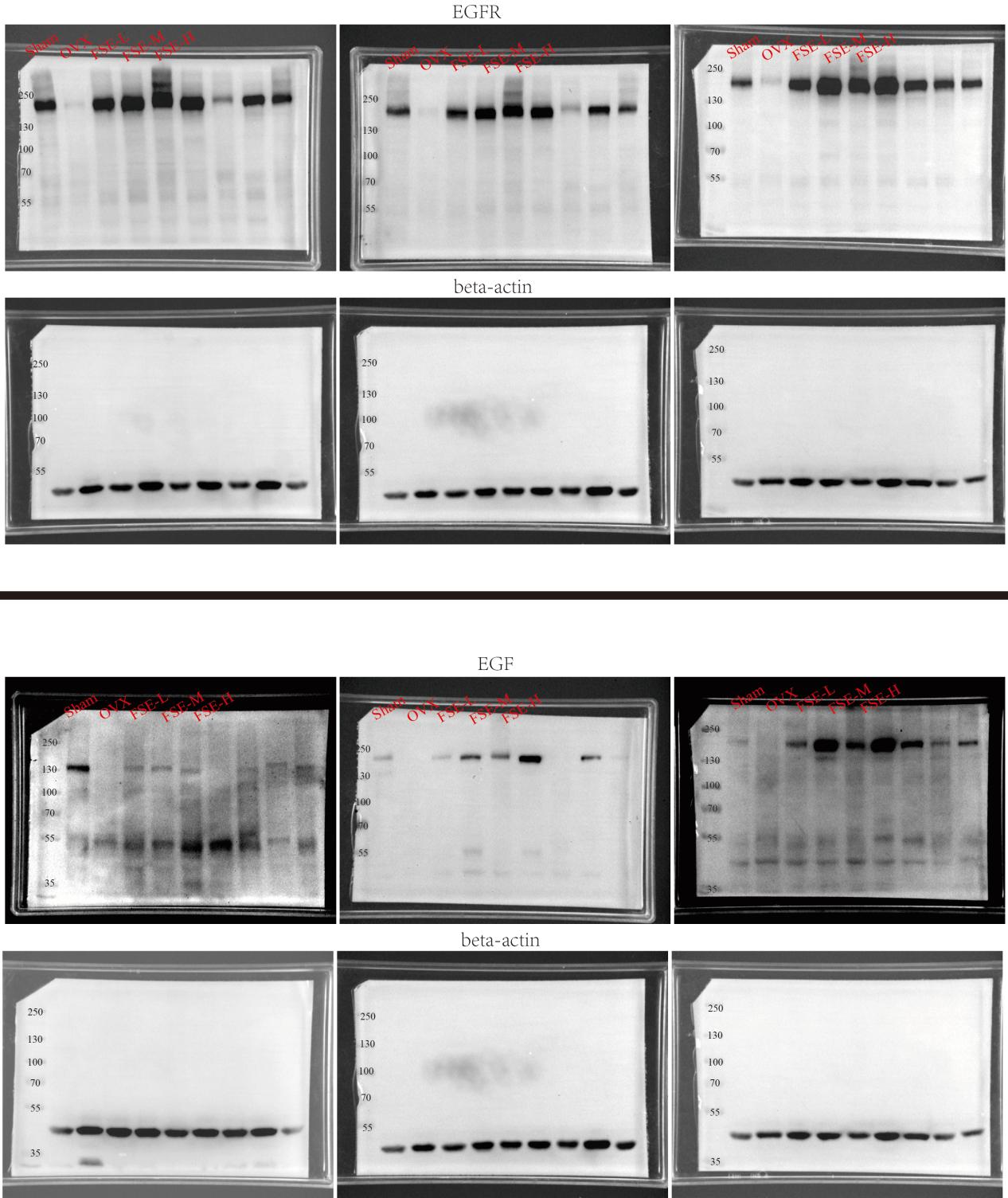

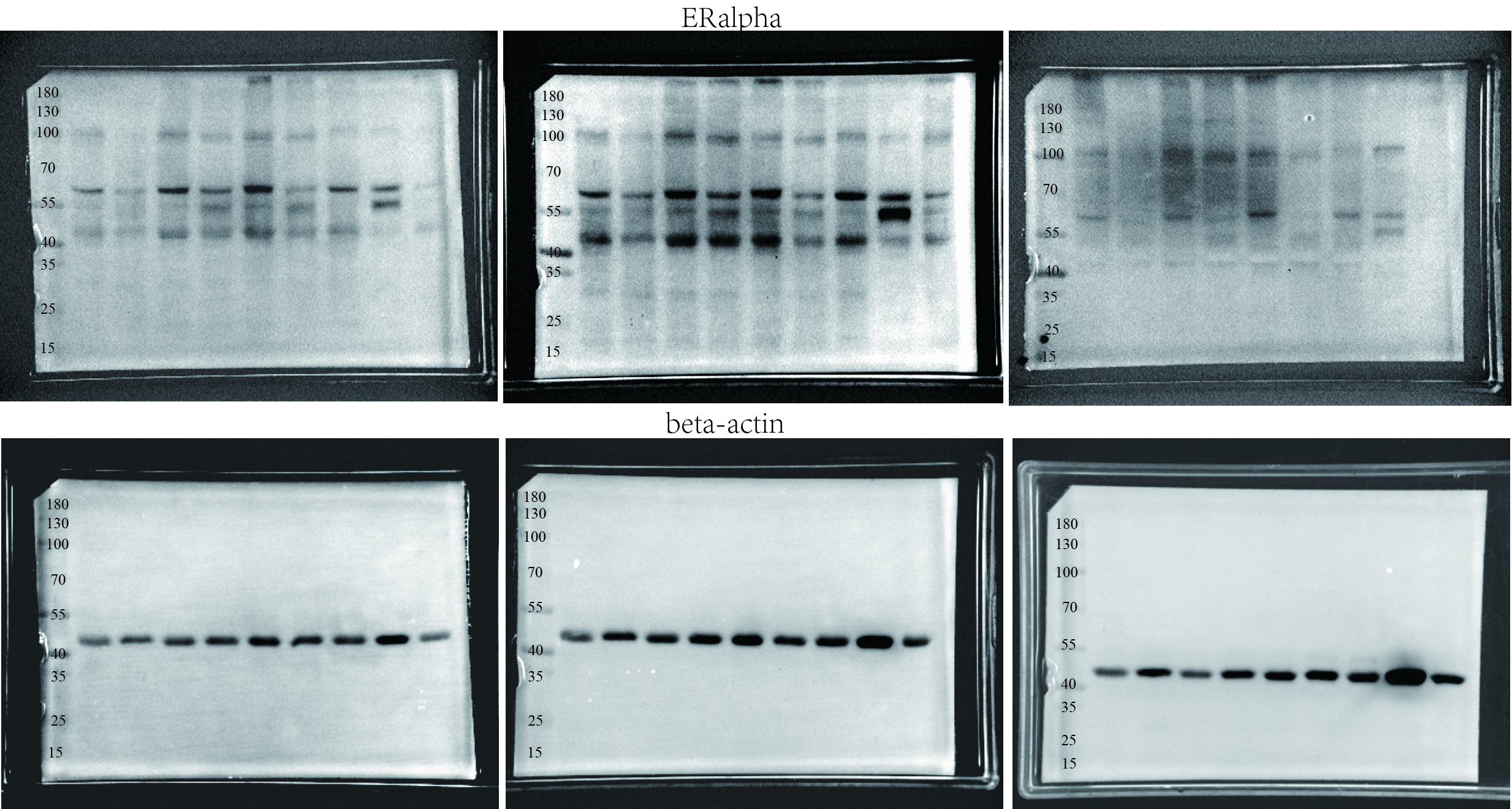


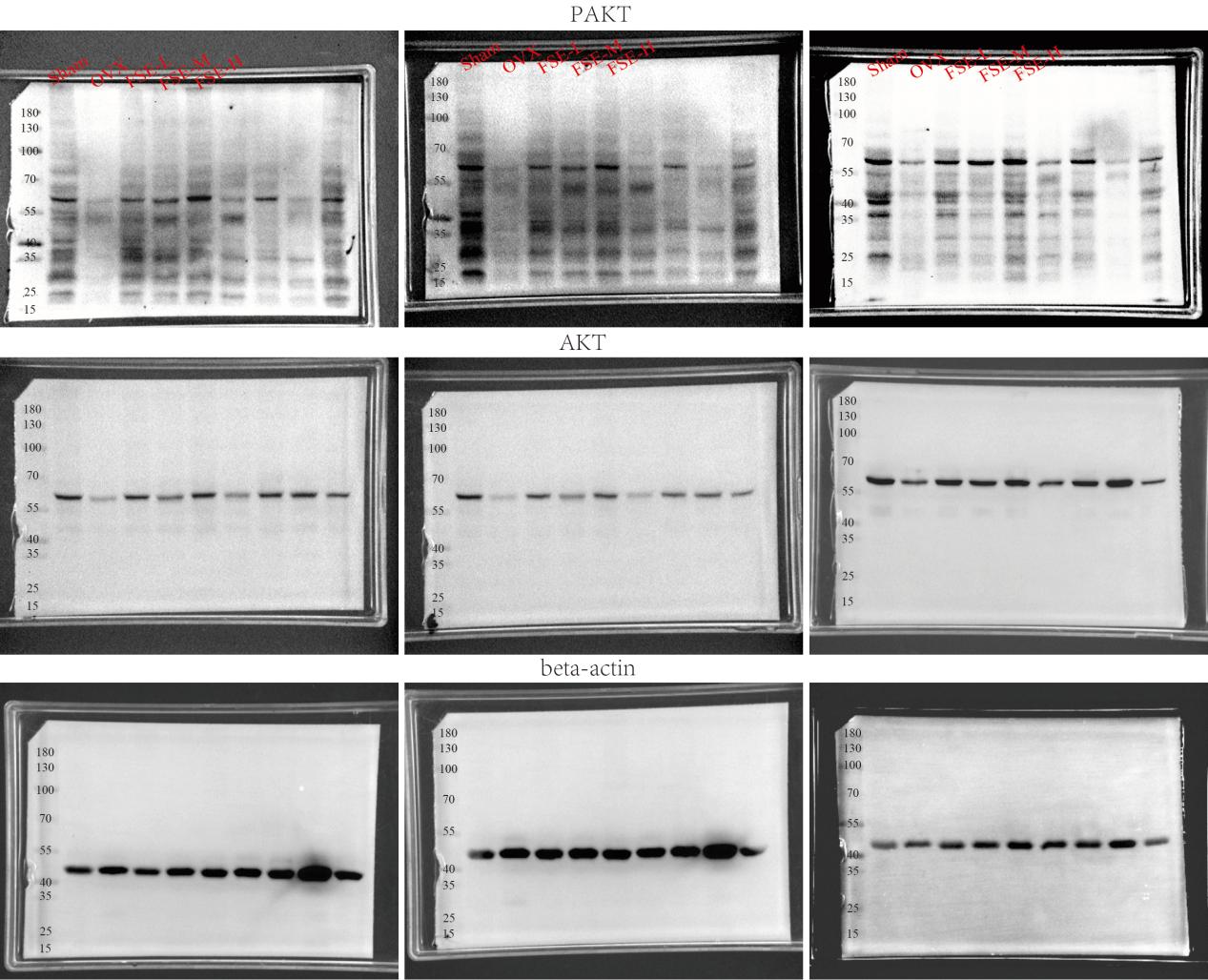

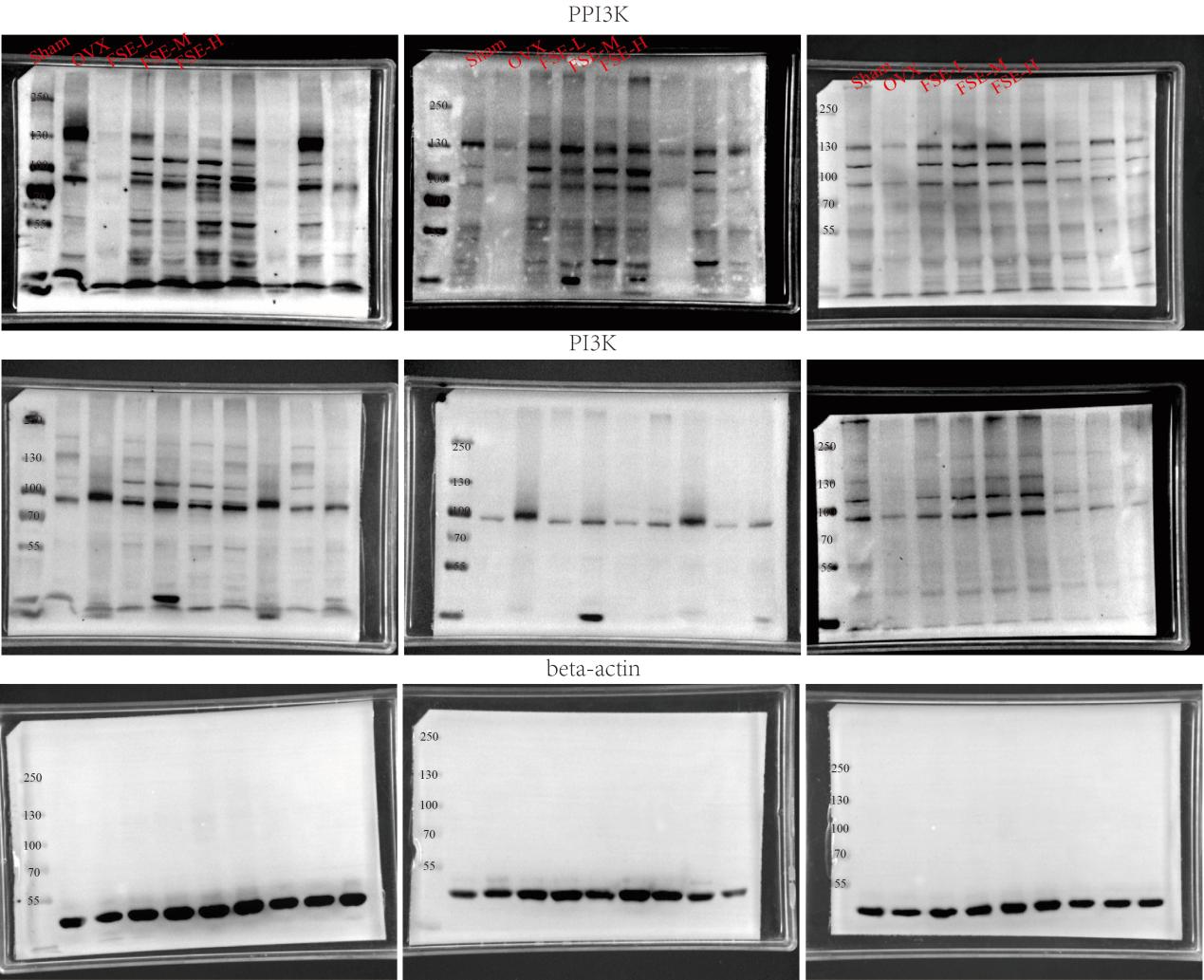

Supplement: Supplementary file 1 [file DataSheet1.zip › original data/Western blot.docx]
